# Otilonium bromide ameliorates paclitaxel-induced peripheral neuropathy by targeting phosphatase PPM1A

**DOI:** 10.1186/s12974-026-03845-9

**Published:** 2026-05-07

**Authors:** Xiaojing Liu, Meng Zhang, Yue Zhang, Yining Hao, Dayun Lu, Wenjun Li, Xu Shen

**Affiliations:** School of Medicine, Nanjing University of Chinese Medicine, Nanjing, 210023 China

**Keywords:** Paclitaxel, Paclitaxel-induced peripheral neuropathy, PPM1A, Macrophage, Inflammation

## Abstract

Paclitaxel (PTX)-induced peripheral neuropathy (PIPN) is a severe side effect lacking effective treatment, largely due to its complex and poorly understood pathogenesis. Here, we observed the pathological inhibition of phosphatase PPM1A activity in the dorsal root ganglia (DRG) tissues of PIPN mice. We also found that otilonium bromide (OB), as a PPM1A activator, ameliorated the PIPN-like pathology in mice, as evidenced by the alleviation of sensory dysfunction, myelin sheath injury, intraepidermal nerve fiber loss and vascular lesions. Using PPM1A-specific knockdown mice, we demonstrated that OB suppresses pro-inflammatory M1 macrophage polarization in the DRG through the PPM1A/NF-κB/NLRP3/IL-1β pathway, thereby alleviating axonal degeneration and neuronal apoptosis. *In vitro* experiments revealed that PTX-damaged DRG neurons release high-mobility group box 1 (HMGB1) to promote pro-inflammatory macrophage polarization, while OB disrupts this neuron-macrophage interaction by limiting HMGB1 release and subsequent macrophage activation. Together, our findings highlight PPM1A activation as a promising therapeutic strategy for PIPN and identify OB as a potential agent for treating this clinical side effect.

**Graphical Abstract:**

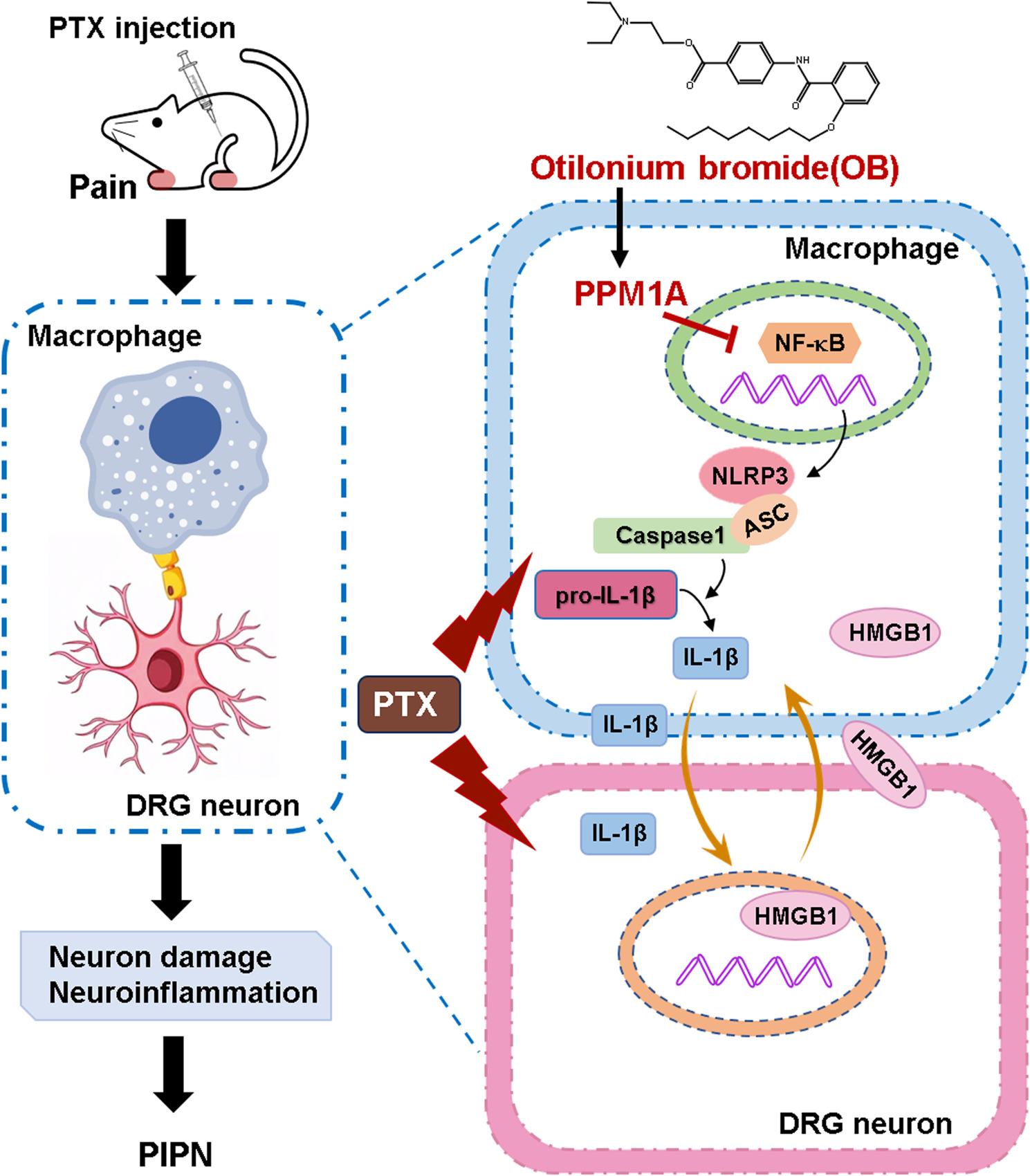

**Supplementary Information:**

The online version contains supplementary material available at 10.1186/s12974-026-03845-9.

## Introduction

Paclitaxel (PTX) as an efficient antineoplastic agent is being widely used for treating a range of solid tumors including breast, endometrial and cervical cancers [1]. 

However, PTX treatment can lead to varied side effects such as peripheral neuropathy, affecting approximately 60–70% of patients [2]. PTX-induced peripheral neuropathy (PIPN) is mainly characterized by sensory disorders with numbness, tingling, hyperalgesia and ectopic pain typically distributed in a stocking-glove pattern [3]. PIPN seriously impairs the quality of life for patients with PTX-based chemotherapy. Owing to the poorly understood pathologies of PIPN, no effective therapeutic strategy has yet been established. Thus, investigating the underlying mechanisms of PIPN and discovering novel therapeutic targets and agents are of great clinical significance.

The dorsal root ganglion (DRG), a cluster of primary sensory afferent neurons adjacent to the intervertebral foramina, serves as a critical relay for transmitting sensory signals into the central nervous system. PTX readily crosses the blood-nerve barrier and accumulates in the DRG, inducing sensory neuron damage and sensory dysfunction [4].

The pathogenesis of PIPN involves multifactorial mechanisms, including neuroinflammation [5], mitochondrial dysfunction [6], apoptosis [7] and axonal degeneration [8] in the peripheral nervous system. Beyond direct neurotoxicity, non-neuronal cells, particularly macrophages, play a crucial role in PIPN progression. PTX upregulates MMP-13 [2] and MCP-1 [9] expression in DRG tissues, promoting macrophage infiltration. These macrophages, characterized by pro-inflammatory markers, exacerbate the sensitization of primary sensory afferent neurons and contribute to neuronal and glial damage. Therefore, this disruption of afferent terminals in the dorsal horn of the spinal cord leads to neuropathic pain [2, 10]. Notably, Toll-like receptor 4 (TLR4) on macrophages drives the polarization of pro-inflammatory M1 macrophages, further amplifying PTX-induced nociceptive effects on DRG neurons [11, 12]. Thus, elucidating the role of macrophage activation is essential for identifying novel therapeutical targets and advancing drug development for PIPN.

Mg^2+^ and Mn^2+^ dependent protein phosphatase 1 A (PPM1A) is widely expressed in various tissues, including the peripheral nervous system [13]. PPM1A is known to negatively regulate stress response, inflammation and apoptosis [14], which are all closely associated with PIPN pathology. It was reported that impaired PPM1A expression during macrophage differentiation impacts cellular adhesion, morphology and inflammatory markers’ expression [15]. Specifically, overexpression of PPM1A promotes an anti-inflammatory phenotype in both THP-1 and human monocyte derived macrophages (hMDMs) [15]. It was noted that inhibition of PTX-induced neuroinflammation and induction of pro-resolving markers including *Arg1* and *Il10* contributed to the alleviation of PIPN symptoms [16], which suggests the beneficial effect of macrophage M2 polarization that is associated with anti-inflammatory response on PIPN pathology. Although the direct evidence linking PPM1A to PTX-induced macrophage polarization has not yet been reported, our current findings reveal that PPM1A plays a critical role in this process.

Given the lack of effective PIPN treatments and the regulatory role of PPM1A in inflammation, we hypothesized that the pathological reduction in PPM1A enzyme activity within DRG contributes to PIPN progression by disrupting macrophage homeostasis. Furthermore, we postulated that restoring PPM1A activity could alleviate neuropathic symptoms. To validate this hypothesis, we first confirmed the suppression of PPM1A activity in PIPN mice. Subsequently, we employed PPM1A-specific knockdown PIPN mice and *in vitro* co-culture assays to investigate whether OB, a well-characterized PPM1A activator, could ameliorate PIPN pathology by modulating macrophage polarization through the PPM1A/NF-κB/NLRP3/IL-1β pathway.

Consequently, the central research question of this study is whether PPM1A serves as a key regulator of neuroinflammation in PIPN, and whether pharmacological activation of PPM1A using OB can effectively alleviate PTX-induced peripheral neurotoxicity without compromising antitumor efficacy.

## Materials and methods

### Reagents

OB (HPLC ≥ 98%, O129950, Aladdin) was dissolved in sterile saline to prepare stock solutions at concentrations of 20 µM or 0.5 mg/mL. PTX (HY-B0015, MedChemExpress) was dissolved in a mixture of Cremophor EL (HY-Y1890, MedChemExpress) and ethanol (10009257, Sinopharm Chemical Reagent Company) before being diluted in sterile saline to achieve a final concentration of 0.4 mg/mL. In the final preparation, the volume ratio of Cremophor EL to ethanol was maintained at 3.3%.

### Animals

Male C57BL/6J mice (7 weeks old) and female NU/NU Nude mice (4–5 weeks old) were purchased from Vital River Laboratory Animal Technology Co., Ltd. All animal experiments were conducted in accordance with the National Institutes of Health guidelines and were approved by the Animal Ethics Committee of Nanjing University of Chinese Medicine (Ethics Registration No. 202106A058; 202204A025). The animals were housed in a specific animal facility under standard conditions, with adequate ventilation and a maintained temperature of 25 °C, following a 12-hour light/dark cycle.

#### PIPN model mice

PIPN was induced in male C57BL/6J mice (PIPN mice) following a previously described protocol [17]. In brief, mice received intraperitoneal (i.p.) injections of PTX (4 mg/kg) on days 1, 3, 5, and 7, for a total of four injections. To minimize pre-injury variability, all mice underwent a one-week habituation period to the test environment. Baseline mechanical thresholds and thermal/cold latencies were measured multiple times before the first PTX injection and only animals exhibiting stable baseline values within the normal physiological range were included in the study [18, 19]. This procedure ensured that any observed behavioral changes were attributable to the PTX exposure rather than pre-existing individual differences. Following the final PTX injection, mice that exhibited a significant reduction in withdrawal thresholds/latencies compared to their individual baselines were identified as PIPN mice. These mice were randomly assigned to experimental groups using a block randomization method to ensure that the average baseline pain sensitivity was balanced across all groups. Researchers were blinded to the group allocations during all behavioral assessments and subsequent data analysis. 

#### PPM1A-specific knockdown mice

PPM1A knockdown (KD) mice were generated by intrathecal (i.t.) injection of adeno-associated virus (AAV)-*Ppm1a*-*shRNA* (WZ Biosciences Inc.) with a viral titer of 1 × 10¹³ vg/mL. The injection was administered into the L4-L6 subarachnoid space of the spinal cord three weeks before the first PTX injection, following the published protocol [20]. The *shRNA* oligonucleotide sequence used for PPM1A knockdown is listed in Table S1 of the Supplementary Materials.

#### Tumor xenograft model mice

MCF-7 cells (3 × 10^7^ cells) expressing the luciferase gene were subcutaneously implanted into the axillary region of female NU/NU nude mice according to a previous report [21]. Seven days later, the mice were randomly assigned to four groups. Tumor growth was monitored on days 0, 7, and 21 post-administrations. Following administration of luciferin, bioluminescence images were captured using a charge-coupled device camera. The luminescence intensity was displayed as heatmaps. Bioluminescence imaging was conducted with an IVIS Lumina XR, and image radiance values were normalized using Living Image software (Perkin Elmer). At the endpoint of the animal experiments, the mice were anesthetized with 5% isoflurane and subsequently sacrificed. 

### Animal administration

Mice were randomly divided into six groups, including vehicle group (Veh), OB treatment group (2.5, 5 mg/kg; OB-2.5, OB-5, i.p.), PIPN group (PTX, 4 mg/kg; PTX-4), PIPN mice treated with OB group (PTX + OB-2.5 and PTX + OB-5) (*n* = 12 per group). OB was administered intraperitoneally once daily for four weeks. OB was administered at 5 mg/kg, as determined by prior pharmacokinetic studies and dose-response optimization [22–25].

AAV-injected mice were randomly divided into five groups, including AAV9-*NC*-injected control group (*NC*-veh), AAV9-*NC*-injected PIPN group (*NC*-PTX), AAV9-*Ppm1a*-*shRNA*-injected control group (*KD*-veh), AAV9-*Ppm1a*-*shRNA*-injected PIPN group (*KD*-PTX), and AAV9-*Ppm1a*-*shRNA*-injected PIPN mice treated with 5 mg/kg OB group (*KD*-PTX + OB) (*n* = 12 per group). Three weeks after AAV injection, PTX (4 mg/kg) was injected intraperitoneally every other day for a total of four times to establish PIPN mice. OB was intraperitoneally injected once a day for four weeks after PIPN was established.

Tumor xenograft mice were randomly divided into vehicle group (Veh), PTX treatment group (PTX), OB treatment group (OB), treatment with PTX and OB group (*n* = 6 per group). Ten days after cell injection, 4 mg/kg PTX was injected i.p. every other day for a total of four times, followed by daily OB or vehicle administration for four weeks.

### Behavioral tests

To assess the initiation and maintenance phases of PIPN, mechanical, thermal, and cold pain sensitivities were evaluated at multiple time points according to the previous study [26]. Testing time points included baseline (prior to the first PTX injection), the early phase (after the last PTX injection) to assess pain initiation and the late phase (weeks 2, 3, 4, and 5) to evaluate pain maintenance and resolution. 

#### Mechanical Withdrawal Threshold (MWT) determination

The sensitivity of mice to mechanical stimulation was assessed using Von Frey filaments (Ugo Basile) according to a published approach [27]. Briefly, mice were acclimated for 15 min in plexiglass boxes (11 × 13 × 24 cm) placed on a metal mesh. Force stimulation ranging from 0.4 to 6.0 g was applied to the plantar surface of the right hind paw of each mouse. A positive response was defined as any of the following behaviors: paw withdrawal, scratching, licking, or raising the paw pad. The formula for calculating the MWT in mice is: 50% threshold = (10^[Xf + kδ])/10000, where Xf is the corresponding force coefficient measured by Von Frey filament, k represents the reference value from the positive/negative reaction parameter table in the Up and Down method, δ = 0.224. 

#### Thermal Withdrawal Latency (TWL) determination

Thermal paw withdrawal latency was measured using a hot/cold plate apparatus (Ugo Basile) to assess thermal sensitivity [17]. Mice were acclimated on the platform for 15 min at room temperature prior to the test. The plate temperature was then set to 50 °C, and the latency to a withdrawal response (such as licking, shaking, or lifting the paw) was recorded. Each mouse was tested three times with 10 min intervals, and the average latency was used for statistical analysis. 

#### Cold Withdrawal Latency (CWL) determination

CWL was measured using the same procedure as for TWL, except that the cold testing apparatus was set to 4 °C [17]. The latency until a withdrawal response was recorded and averaged from at least three trials per mouse. 

#### Conditioned Place Preference (CPP) determination

Spontaneous pain was assessed using the CPP test following an established protocol [28]. The CPP apparatus comprised two compartments (15 × 18 × 20 cm) connected by an intermediate compartment (15 × 8 × 20 cm). The left compartment had black walls and a striped floor, whereas the right compartment had white walls and a mesh floor. To confine the animal to a specific compartment during the training, removable barriers were installed. During preconditioning, mice were placed in the central chamber and allowed to explore all three chambers freely for 15 min. Video tracking was used to record the time spent in each chamber to establish baseline preferences. Mice showing extreme chamber preference (> 80% or < 20% of total time) were excluded. For the remaining mice, the less preferred chamber was designated as the drug-paired side.

Conditioning occurred over two days. Mice received an i.p. injection of saline in the morning and were confined to the saline-paired chamber for 30 min. Four hours later, they received an i.p. injection of OB (5 mg/kg) and were confined to the drug-paired chamber for 30 min. On the day of post-conditioning, mice had free access to all chambers for 15 min, and the time spent in each chamber was recorded.

#### Open field test

Locomotor activity was assessed using the open field test in an arena (50 × 50 cm) [29]. Mice were habituated to the test environment prior to the experiment. On the test day, each mouse was placed in the center of the arena and allowed to explore freely for 10 min with behavior recorded on video for subsequent analysis. 

#### Rotarod test

Motor coordination was assessed to evaluate potential OB neurotoxicity [19, 29]. Mice were placed on a rotarod that accelerated from 4 to 40 rpm over a 5-min period, and the latency to fall was recorded, with a maximum cut-off time of 300 s. Mice underwent three training sessions 24 h prior to the first test to familiarize them with the procedure, with a 30-min interval between each session. 

### Blood flow velocity measurement

Mice were anesthetized using an animal anesthesia machine with isoflurane and Laser speckle contrast imaging (LSCI; RFLSI Pro, RWD) was used to detect the real-time local blood flow velocity of foot pads and sciatic nerve tissues of mice [30]. Blue areas represented the low blood flow velocity, and red areas represented the high flow velocity. Images were analyzed and quantified using ImageJ software.

### Cell culture

#### Primary DRG neuron preparation

DRG tissues were aseptically dissected from mice (8 weeks old) and digested with collagenase I (1.14 mg/mL, C0130, Sigma-Aldrich) and dispase I (2.15 mg/mL, 17105041, Invitrogen) at 37 °C for 30 min. Primary DRG neurons were isolated and plated onto the culture plates pre-coated with poly-D-lysine and laminin. The cells were cultured in Ham’s F-12 medium (10-080-CV, Corning) supplemented with 2% B27 (17504044, Invitrogen), 1% penicillin and streptomycin (P/S, BC-CE-007, Bio-channe) [31]. 

#### Bone Marrow-Derived Macrophage (BMDM) preparation

BMDMs were isolated from the tibiae and femora of normal mice. Bone marrow was flushed with cold PBS, and the collected cells were dissociated, filtered through a 70 μm strainer, and treated with red blood cell lysis buffer. After centrifugation, the cells were finally resuspended and cultured in RPMI 1640 medium (10-040-CV, Corning) supplemented with 10% fetal bovine serum (FBS, 10099-141, Gibco Grand Island), 1% P/S, and M-CSF (abs04383, Absin) for differentiation [32]. 

#### Cell line culture

RAW264.7 cells and MCF-7 human breast cancer cells were cultured in Dulbecco’s modified Eagle’s Minimum Essential Medium (DMEM, BC-M-005, Biochannel) supplemented with 10% FBS and 1% P/S [17, 33].

All cells were maintained in a humidified incubator with 5% CO_2_ at 37 °C. 

### Immunofluorescence

#### Tissue-based assay

The DRG and foot pad tissues were fixed in 4% paraformaldehyde, whereas the sciatic nerve tissues were fixed in 2.5% glutaraldehyde for 24 h. After dehydration in 30% sucrose, tissues were sectioned into 5 μm slices using a cryostat (Leica) and mounted on slides. 

#### Cell-based assay

Primary DRG neurons were seeded on slides and incubated with PTX (0.1 µM) and/or OB (1 or 5 µM) for 24 h. BMDMs were plated onto slides and incubated with PTX (5 µM) and/or OB (1 or 5 µM) for 3.5 h. ATP (3 mM) was then added for an additional 0.5 h. Cells were fixed in 4% paraformaldehyde for 15 min before proceeding to immunofluorescence staining.

All slides were permeabilized with Triton X-100 for 10 min, blocked with 10% goat serum for 1 h, and incubated with primary antibodies overnight at 4 °C, followed by corresponding secondary antibodies for 2 h at room temperature. Nuclei were stained with Hoechst 33,342 for 5 min, and images were acquired using a fluorescence microscope (Leica) and analyzed with ImageJ [17]. Antibodies are listed in Table S2 of the Supplementary Materials.

### Total neurite outgrowth determination

DRG neurons isolated from mice were seeded in 12-well plates overnight, fixed with 4% paraformaldehyde, and then permeabilized with Triton X-100 and blocked with 4% bovine serum albumin (BSA, A8020, Solarbio). The neurons were incubated with anti-β-tubulin III antibody (1:1 000; T8578, Sigma-Aldrich) overnight at 4 °C, followed by Alexa Fluor 488-conjugated goat anti-mouse IgG (H + L) (1:250, SA00013-1, Proteintech) for 1 h at room temperature. Neurite growth was visualized and measured using a fluorescence microscope and ImageJ [31].

### TUNEL assay

The DRG neurons from normal mice were seeded in 24-well plates and treated with PTX (0.1 µM) and/or OB (1 or 5 µM) for 24 h. Apoptosis of DRG neurons was detected using a TUNEL apoptosis assay kit (C1090, Beyotime), following the manufacturer’s instructions. The cells were visualized using a fluorescence microscope and quantified by ImageJ.

### RNA extraction and quantitative real-time PCR

Total RNA was extracted from primary cells or tissues using Trizol reagent (TKR-9109, Takara) [34]. Briefly, 1 µg of total RNA was reverse transcribed into cDNA using the HiScript III RT SuperMix (R323-01, Vazyme). Quantitative real-time PCR (qPCR) was performed to assess the mRNA levels of various genes using the SYBR Premix Ex Taq kit (Q321-02, Vazyme). The expression levels were normalized to β-actin. All primers were custom synthesized by Sangon Biotech (Shanghai) and their sequences are detailed in Table S3 of the Supplementary Materials.

### MTT assay

MCF-7 cells were seeded in 96-well plates at a density of 1 × 10^4^ cells/well and cultured overnight, followed by incubation with 0.1 µM PTX and/or 5 µM OB for 24 h. After 24 h of incubation, 0.5 mg/mL MTT solution (M2128, Sigma-Aldrich) was added to each well, and the plates were incubated for 4 h at 37 °C. Formazan crystals were then dissolved in 100 µL of dimethyl sulfoxide (DMSO, 219605580, MP Biomedicals). The plates were shaken for 15 min at room temperature to ensure complete dissolution of the crystals. The absorbance was measured at 490 nm using a microplate reader (Molecular Devices, Silicon Valley) to assess cell viability.

### Conditioned medium assay

#### BMDMs were cultured with supernatants from primary DRG neurons

After PTX stimulation of primary DRG neurons for 24 h, the conditioned medium (supernatant, abbreviated as CM) was collected and transferred to BMDMs, which had been pretreated with OB for 24 h. After the treatment, the original medium was discarded, and the cells were incubated with the collected DRG neuron-CM for an additional 24 h to assess the effects of DRG neuron secretions on BMDMs. 

#### Primary DRG neurons were cultured with supernatants of BMDMs

BMDMs were pretreated with OB for 24 h, followed by stimulation with PTX for 3.5 h. After PTX stimulation, ATP was added for an additional 30 min. The CM was then collected and applied to culture primary DRG neurons, which were incubated with the medium for another 24 h to assess the effects of BMDM secretions on neurons.

### Western blot

#### Tissue-based assay

Tissue samples were homogenized and lysed in RIPA buffer (P0013D, Beyotime) containing protease and phosphatase inhibitors for 30 min. 

#### Cell-based assay

Cells were lysed with RIPA buffer containing protease and phosphatase inhibitors on ice for 30 min.

All lysates were centrifuged at 12000 rpm for 30 min at 4 °C, and the supernatant was collected. Protein concentration was determined using a BCA assay kit (P0009, Beyotime). Proteins were separated by SDS-PAGE and transferred to nitrocellulose membranes (10600003, GE Whatman). After blocking with 5% milk for 2 h, membranes were incubated overnight at 4 °C with primary antibodies (1:1000), followed by secondary antibodies (1:3000) for 2 h at room temperature. Protein bands were visualized using a ChemiDoc MP imaging system (Bio-Rad, California, USA) and quantified with ImageJ [31]. Antibodies are listed in Table S4 of the Supplementary Materials.

### Enzyme-Linked Immunosorbent Assay (ELISA)

The IL-1β concentration in the supernatant of BMDMs was measured using a mouse IL-1β ELISA kit (VAL601, Novus Biologicals) according to the manufacturer’s instructions. Briefly, the supernatant was centrifuged, and samples/standards were added to the plate followed by a 2 h incubation. After washing, the detection conjugate was added and incubated for another 2 h. Subsequently, the plate was incubated with TMB substrate for 30 min in the dark before the reaction was stopped, and the absorbance was measured at 450 nm.

### siRNA transfection

RAW264.7 cells were transiently transfected with 50 nM siRNA (si-*NC*) or *Ppm1a*-siRNA (si-*Ppm1a*) (Genomeditech) using Lipofectamine 2000 transfection reagent (11668019, Invitrogen) according to the manufacturer’s instructions. Six hours after transfection, the medium was replaced with complete DMEM medium and cells were cultured for an additional 24 h. The knockdown efficiency was assessed using western blot assay [24].

### Co-Immunoprecipitation (co-IP)

Macrophages were collected and lysed in NP-40 buffer on ice for 30 min. The supernatants were incubated with anti-NF-κB antibody overnight at 4 °C, followed by incubation with Protein G agarose for an additional 3 h at 4 °C. The obtained protein complexes were prepared for western blot assay.

### Statistical analysis

All data were expressed as mean ± SEM and analyzed by GraphPad Prism version 8.0.1. Details of the statistical analysis and the sample size for each experiment are indicated in the figure legends. Unpaired two-tailed Student’s t-test was used for comparisons between two groups. One-way or two-way ANOVA followed by Dunnett’s post hoc test was used for multiple group comparisons as indicated in the figure legends. Statistical significance was defined as *P* < 0.05.

## Results

### PPM1A enzyme activity is repressed in DRG tissues of PIPN mice

**Fig. 1:**
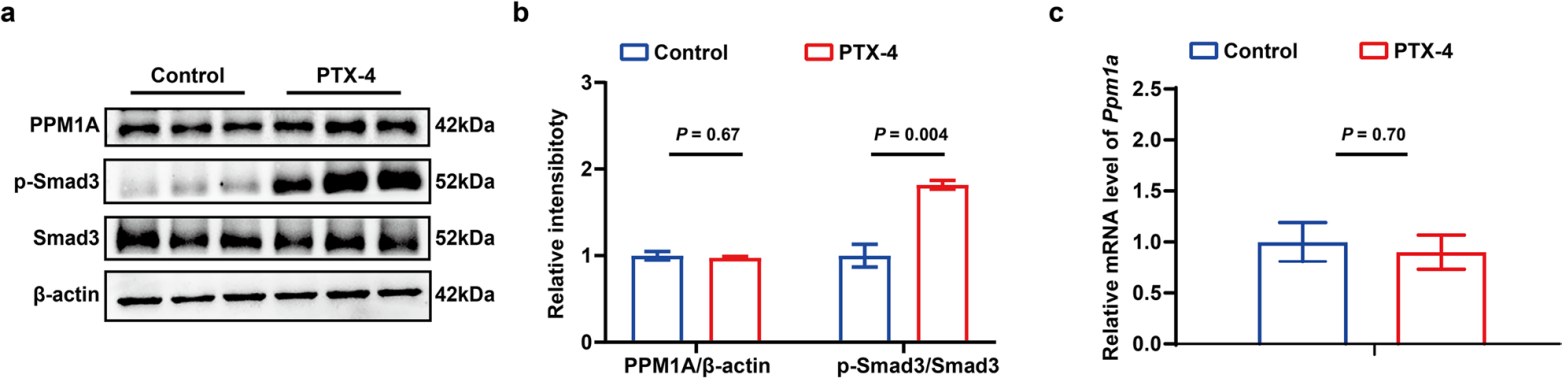
PPM1A enzyme activity is repressed in DRG tissues of PIPN mice.** a**,** b** Western blot analysis and corresponding quantification of PPM1A, phosphorylated Smad3 (p-Smad3) and Smad3 protein levels in dorsal root ganglion (DRG) tissues of PIPN mice (*n* = 3 per group). **c** qPCR analysis of *Ppm1a* mRNA level in DRG tissues of PIPN mice (*n* = 10–12 per group). All values are presented as mean ± SEM. Statistical significance was determined using Student’s t-test. Exact P values are shown above the bars

PPM1A is a Mg^2+^/Mn^2+^-dependent protein phosphatase that plays a critical role in inflammation regulation, stress response and apoptosis, which are all implicated in the pathogenesis of PIPN [14]. To investigate whether PPM1A expression or activity is pathologically altered in PIPN, Western blot and qPCR assays were carried out in DRG tissues of PIPN mice. Western blot results revealed that the PPM1A protein level remained unchanged, while the p-Smad3 level was elevated compared to the control group (*P* = 0.67, *P* = 0.004, respectively; Fig. 1a and b). Given that p-Smad3 is a specific substrate of PPM1A and a marker of PPM1A phosphatase activity [14], this finding indicates the suppression of PPM1A activity. In addition, qPCR results also confirmed that *Ppm1a* mRNA levels were unaltered in the DRG tissues of PIPN mice (*P* = 0.70; Fig. 1c).

All results demonstrate that PPM1A enzyme activity, rather than its protein level, is repressed in DRG tissues of PIPN mice.

### OB as a PPM1A activator alleviates peripheral neuropathy in PIPN mice by targeting PPM1A

**Fig. 2:**
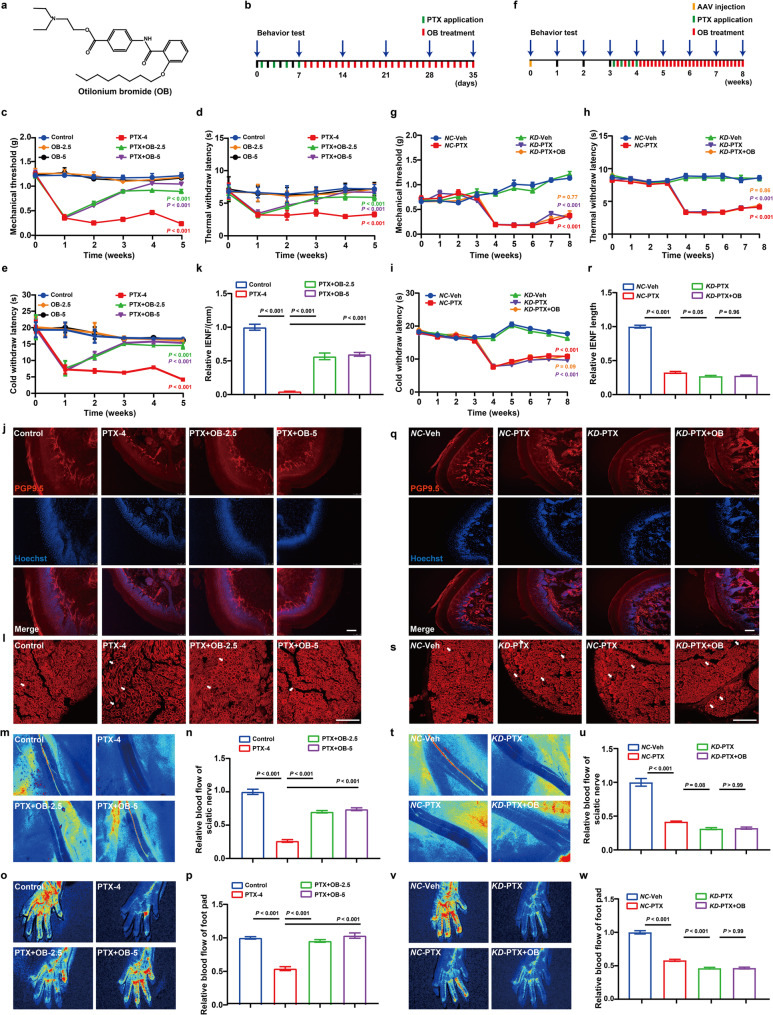
OB as a PPM1A activator alleviates peripheral neuropathy in PIPN mice. **a** Chemical structure of otilonium bromide (OB). **b** Schedule of animal treatments and behavioral tests. **c** Mechanical threshold assay in PIPN mice (*n* = 12 per group). **d** Thermal response assay in PIPN mice (*n* = 12 per group). **e** Paw cold response assay in PIPN mice (*n* = 12 per group). **f** Schedule of animal treatments and behavioral tests of AAV-*Ppm1a*-*shRNA*-injected PIPN mice (KD). **g** Mechanical threshold assay in AAV-*Ppm1a*-*shRNA*-injected PIPN mice (*n* = 12 per group). Paw response latency to (**h**) thermal and (**i**) cold stimuli of PIPN mice injected with AAV-Ppm1a-shRNA (n = 12 per group). **j**, **k** Representative immunofluorescence images with quantification of intraepidermal nerve fibers (IENFs, labeled by PGP9.5, red) in foot pads of PIPN mice (n = 4 per group). Nuclei are stained with Hoechst (blue). Scale bar: 50 μm. **l** Representative immunofluorescence images of myelin sheath (labeled by MBP, red) in sciatic nerves of PIPN mice (n = 4 per group). Scale bars: 50 μm. Representative images with quantification of the blood flow velocity in (**m**, **n**) sciatic nerves and (**o**, **p**) foot pads of PIPN mice (n = 4 per group). **q**, **r** Representative immunofluorescence images with quantification of IENFs (labeled by PGP9.5, red) in foot pads of AAV-Ppm1a-shRNA-injected PIPN mice (n = 4 per group). Nuclei are stained with Hoechst (blue). Scale bar: 50 μm. **s** Representative immunofluorescence images of myelin sheath (labeled by MBP, red) in sciatic nerves of AAV-Ppm1a-shRNA-injected PIPN mice (n = 4 per group). Scale bar: 50 μm. Representative images with quantifications of the blood flow velocity in (**t**, **u**) sciatic nerves and (**v**, **w**) foot pads of AAV-Ppm1a-shRNA-injected PIPN mice (n = 4 per group). All values are presented as mean ± SEM. Statistical significance was determined using one-way ANOVA followed by Dunnett’s post hoc test. Exact P values are shown above the bars

Given the observed reduction in PPM1A enzyme activity in DRG tissues of PIPN mice, we next investigated whether pharmacological activation of PPM1A by OB (Fig. 2a), a PPM1A activator [24], could ameliorate PIPN-like symptoms in mice. In the assay, OB was administered at a dose of 2.5 or 5 mg/kg as previously described [24] to assess its efficacy in improving peripheral neuropathic features including hyperalgesia, intraepidermal nerve fiber (IENF) loss, microvascular dysfunction and DRG neuronal damage in PIPN mice.

#### OB improves pain hypersensitivity in PIPN mice by targeting PPM1A

The schedule of behavioral tests is shown in Fig. 2b. Behavioral test results confirmed the development of neuropathic pain in PIPN mice, as demonstrated by reduction in the 50% mechanical threshold (*P* < 0.001, vs. Control group; Fig. 2c), and shortened paw withdrawal latencies in response to both thermal (*P* < 0.001, vs. Control group; Fig. 2d) and cold stimuli (*P* < 0.001, vs. Control group; Fig. 2e) compared to control mice. Notably, OB treatment alleviated pain hypersensitivity in PIPN mice, as evidenced by increased mechanical withdrawal thresholds (*P* < 0.001, vs. PTX group; Fig. 2c) and prolonged paw withdrawal latencies to thermal (*P* < 0.001, vs. PTX group; Fig. 2d) and cold stimuli (*P* < 0.001, vs. PTX group; Fig. 2e). Moreover, OB treatment had no impact on body weight (*P* = 0.74, vs. PTX group; Fig. S1a) or food intake in mice throughout the experimental period (*P* = 0.98, vs. PTX group; Fig. S1b).

To investigate whether the analgesic effects of OB are mediated specifically through targeting PPM1A, we performed a specific knockdown of *Ppm1a* in the peripheral nervous system in PIPN mice by using intrathecal injection of AAV9-*Ppm1a*-*shRNA*. The knockdown efficiency of *Ppm1a* in DRG tissues was measured by western blot assay three weeks post injection (*P* < 0.001; Fig. S2a and b). Behavioral tests were conducted according to the schedule shown in Fig. 2f. The results demonstrated that the alleviative effects of OB treatment (5 mg/kg) on mechanical hyperalgesia (*P* = 0.77, vs. *KD*-PTX group; Fig. 2g), thermal hypersensitivity (*P* = 0.86, vs. *KD*-PTX group; Fig. 2h) and cold allodynia (*P* = 0.09, vs. *KD*-PTX group; Fig. 2i) were abolished in *Ppm1a*-knockdown PIPN mice.

Collectively, these results indicate that OB relieves pain hypersensitivity in PIPN mice by targeting PPM1A.

#### OB ameliorates peripheral nerve structural damage and vascular impairment in PIPN mice by targeting PPM1A

Immunofluorescence assay results demonstrated that OB increased the IENFs density (labeled by PGP9.5 [35], red) in the foot pads (*P* < 0.001 for all; Fig. 2j and k), and reduced myelin sheath damage (labeled by MBP [36], red) accompanied by decreased demyelination, axon degeneration, and vacuole formation in the sciatic nerves of PIPN mice (Fig. 2l). Additionally, the LSCI assay results revealed that PTX treatment led to a reduction of blood flow velocity in both the sciatic nerves (*P* < 0.001 for all; Fig. 2m and n) and foot pads (*P* < 0.001 for all; Fig. 2o and p) of PIPN mice, whereas OB treatment restored this reduction, indicative of its ameliorative effect on microvascular function (*P* < 0.001 for all; Fig. 2m-p).

To determine whether these structural and vascular improvements mediated by OB were dependent on PPM1A, we assessed the related effects of OB in PIPN mice with *Ppm1a* knockdown. Immunofluorescence results revealed that OB lost its restorative effect on IENF density in foot pads (*P* = 0.96; Fig. 2q and r) and myelin damage in sciatic nerves (Fig. 2s) of *Ppm1a*-knockdown mice. Similarly, LSCI assay results also showed that OB failed to reverse the PTX-induced reduction in blood flow velocity in the sciatic nerves (*P* < 0.001, *P* > 0.99, respectively; Fig. 2t and u) and foot pads (*P* < 0.001, *P* > 0.99, respectively; Fig. 2v and w) in AAV-*Ppm1a*-*shRNA*-treated PIPN mice.

Together, these results indicate that OB ameliorates peripheral nerve structural damage and vascular injury in PIPN mice by targeting PPM1A.

#### OB protects DRG neurons from PTX-induced injury by targeting PPM1A

Considering that PTX-induced neuropathic pain is primarily attributed to sensory neuronal damage (e.g. axonal degeneration) in DRG neuronS [37, 38], we next evaluated the impact of OB on neurite outgrowth in DRG tissues from PIPN mice.

**Fig. 3:**
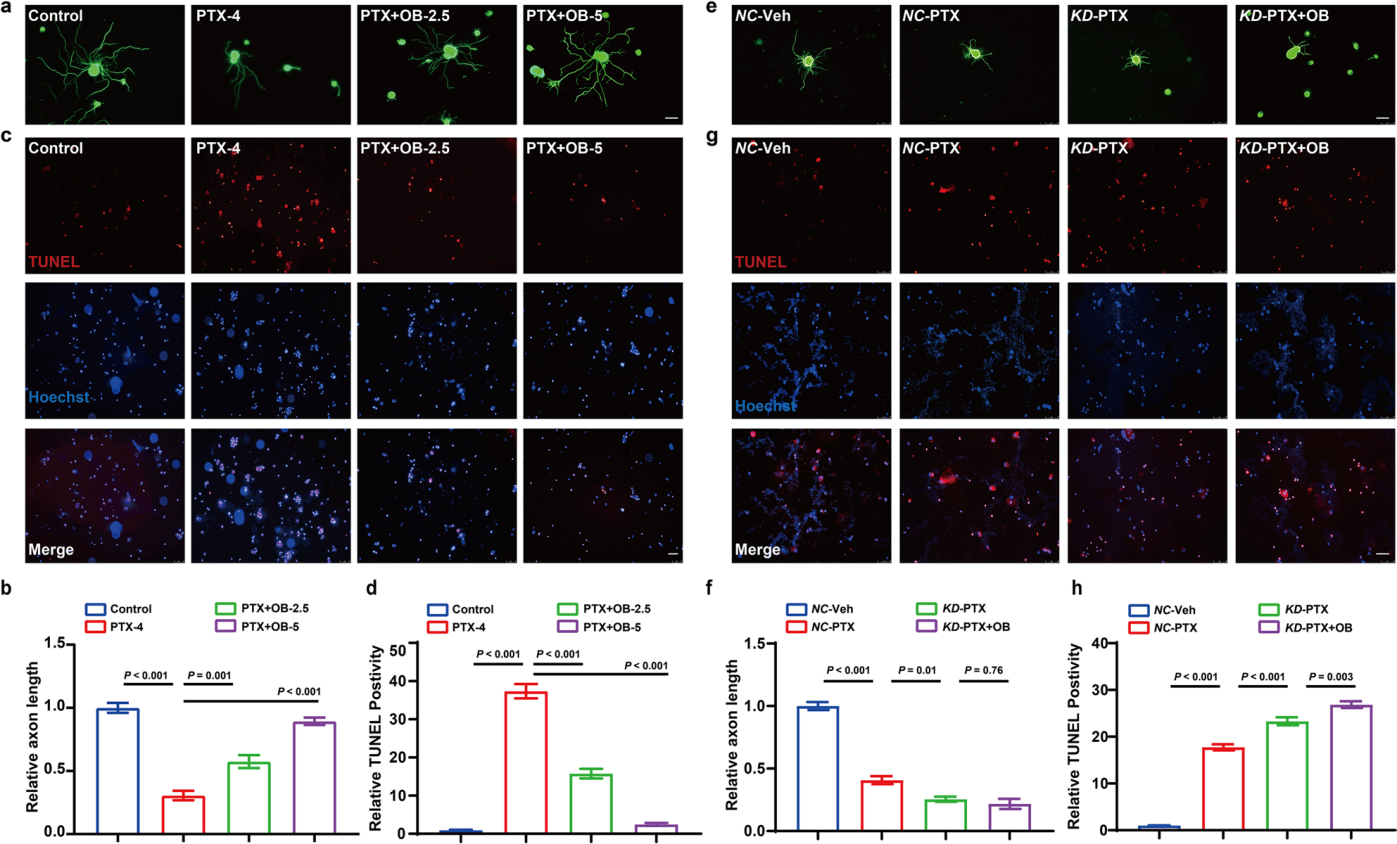
OB protects DRG neurons in PIPN mice by PPM1A.** a**,** b** Representative immunofluorescence images with quantification of total neurite length in DRG neurons (labeled by β-tubulin III, green) of PIPN mice (*n* = 4 per group). Scale bars: 50 μm. **c**,** d** TUNEL assay detecting apoptosis in DRG tissues of PIPN mice (*n* = 4 per group). Scale bar: 75 μm. **e**,** f** Representative immunofluorescence images with quantification of total neurite length of DRG neurons (labeled by β-tubulin III, green) in AAV-*Ppm1a*-*shRNA*-injected PIPN mice (*n* = 4 per group). Scale bar: 50 μm. **g**,** h** TUNEL assay detecting apoptosis in DRG tissues of AAV-*Ppm1a*-*shRNA*-injected PIPN mice (*n* = 4 per group). Scale bar: 50 μm. All values are presented as mean ± SEM. Statistical significance was determined using one-way ANOVA followed by Dunnett’s *post hoc* test. Exact P values are shown above the bars

Immunofluorescence assay results revealed that the total neurite length was reduced in PIPN mice compared to controls (*P* < 0.001), and this reduction was ameliorated by OB treatment (*P* < 0.001; Fig. 3a and b). Additionally, TUNEL assay results demonstrated that OB attenuated neuronal apoptosis in the DRG of PIPN mice (*P* < 0.001 for all; Fig. 3c and d). Importantly, these protective effects on both neurite outgrowth (*P* = 0.76; Fig. 3e and f) and neuronal apoptosis (*P* = 0.003; Fig. 3g and h) were lost in the AAV-*Ppm1a*-*shRNA*-treated PIPN mice.

Together, these results demonstrate that OB as a PPM1A activator attenuates peripheral neuropathy in PIPN mice by restoring sensory function, protecting neuronal structure, preserving microvascular perfusion and preventing neuronal degeneration by targeting PPM1A.

### OB shows no CNS toxicity and impact on spontaneous pain

#### OB treatment lacks CNS toxicity

To evaluate the potential CNS effects of systemic OB, we assessed spontaneous locomotor activity and motor coordination in PIPN mice following intraperitoneal injection of 5 mg/kg OB (the maximum therapeutic dose used in this study) as previously described [29]. In the open field test, OB treatment did not alter exploratory behavior, as evidenced by the lack of significant differences in total distance traveled (*P* = 0.54; Fig. S3a-b) or average speed (*P* = 0.54; Fig. S3c) compared to baseline. In addition, the rotarod test results showed that OB treatment did not impair the motor coordination of PIPN mice (*P* = 0.77, *P* = 0.91, respectively; Fig. S3d). These findings demonstrate that OB is devoid of CNS-associated behavioral toxicity at its effective dose.

#### OB treatment shows no impact on spontaneous pain

Furthermore, we employed the CPP test to assess whether OB alleviates spontaneous pain in PIPN mice [28].

As shown in Fig. S4a and b, PIPN mice treated with OB (5 mg/kg, i.p.) failed to exhibit a significant preference for the drug-paired chamber (*P* = 0.13, *P* = 0.41, respectively). This outcome stands in contrast to traditional analgesics, which typically induce a strong place preference. These results indicate that OB treatment may not suppress the spontaneous pain under our experimental conditions.

In summary, these results demonstrate that systemic OB treatment at the therapeutic dose is devoid of CNS-associated behavioral toxicity and does not induce any rewarding or place-preference effects in PIPN mice.

### OB alleviates NLRP3 inflammasome-mediated macrophage M1 polarization in PIPN mice by targeting PPM1A

As indicated in the published reports, monocyte infiltration into the DRG followed by differentiation into macrophages and the expansion of resident macrophage populations plays a crucial role in initiating and maintaining neuropathic pain under various pathological conditions [16, 39–41]. Persistent activation of these macrophages has been implicated in the development of PIPN [16]. Given the relevance of macrophage inflammatory activation to PIPN pathogenesis, we next investigated whether OB modulates macrophage polarization and neuroinflammation in DRG tissues of PIPN mice. 

#### OB attenuates macrophage infiltration and M1 polarization of DRG in PIPN mice by targeting PPM1A

**Fig. 4:**
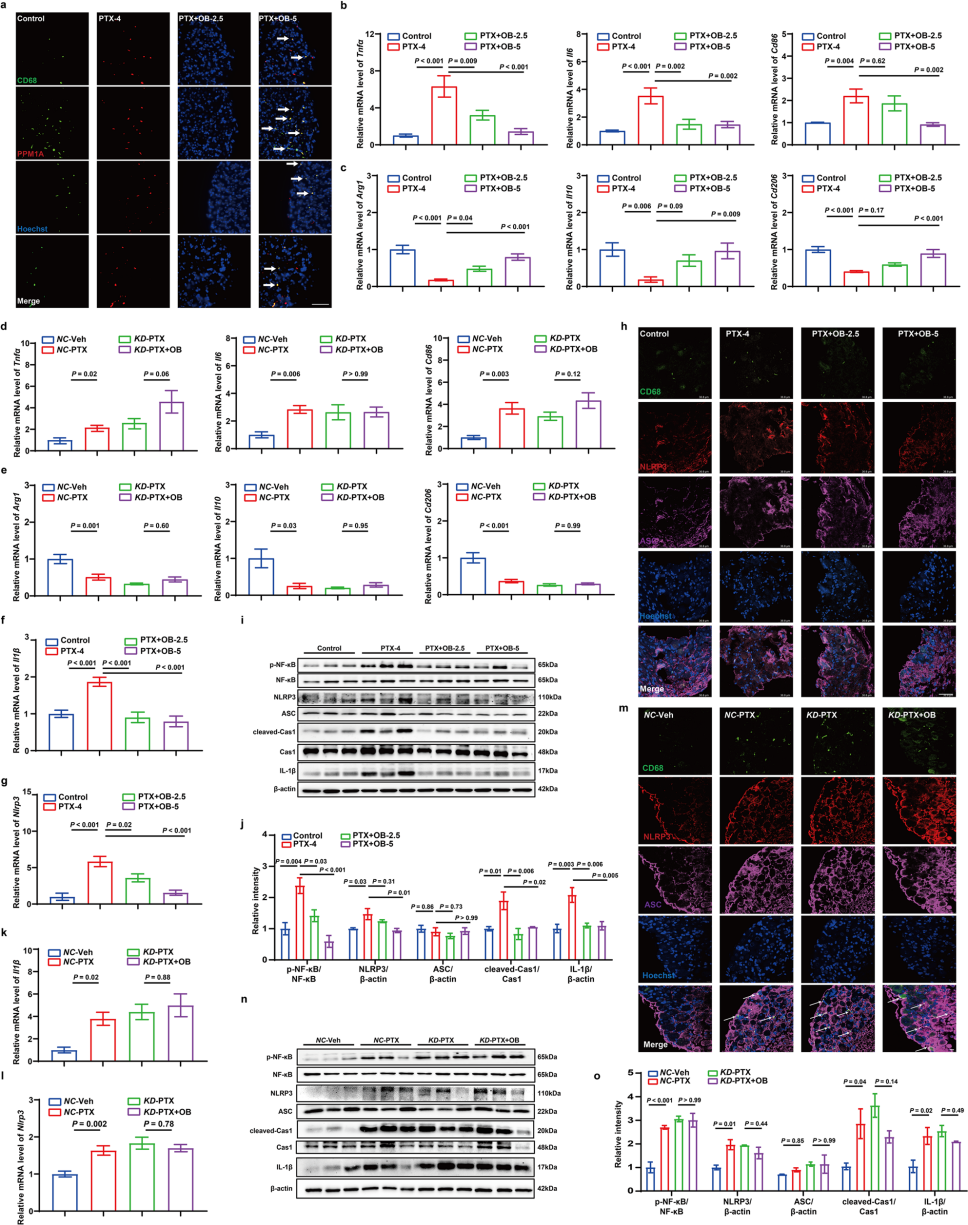
OB attenuates macrophage M1 polarization via PPM1A/NF-κB/NLRP3 pathway.** a** Representative immunofluorescence images of CD68^+^ macrophages (green) in DRG tissues of PIPN mice (*n* = 5 per group). Scale bars: 50 μm. qPCR analysis of mRNA levels of **(b)** M1 (*Tnfα*, *Il6* and *Cd86*) and **(c)** M2 macrophage markers (*Arg1*, *Il10* and *Cd206*) in DRG tissues of PIPN mice (*n* = 6 per group). **d** qPCR analysis of mRNA levels of M1 (*Tnfα*, *Il6* and *Cd86*) and **(e)** M2 macrophage markers (*Arg1*, *Il10* and *Cd206*) in DRG tissues of AAV-*Ppm1a*-*shRNA*-injected PIPN mice (*n* = 6 per group). qPCR analysis of mRNA levels of **(f)** *Il1β* and **(g)** *Nlrp3* in DRG tissues of PIPN mice (*n* = 8 per group). **h** Representative immunofluorescence images of CD68 (green), NLRP3 (red) and ASC (purple) in DRG tissues of PIPN mice (*n* = 5 per group). Scale bar: 36.8 μm. **i**,** j** Western blot analysis and quantification of phosphorylated NF-κB (p-NF-κB), total NF-κB, NLRP3, ASC, cleaved-Cas1, Caspase-1 (Cas1), and IL-1β protein levels in DRG tissues of PIPN mice (*n* = 3 per group). qPCR analysis of mRNA levels of **(k)** *Il1β* and **(l)** *Nlrp3* in DRG tissues of AAV-*Ppm1a*-*shRNA*-injected PIPN mice (*n* = 8 per group). **m** Representative immunofluorescence images of macrophages (labeled by CD68, green), NLRP3 (red) and ASC (purple) in DRG tissues of AAV-*Ppm1a*-*shRNA*-injected PIPN mice (*n* = 4 per group). Scale bar: 10 μm. **n**,** o** Western blot analysis and quantification of p-NF-κB, NF-κB, NLRP3, ASC, cleaved-Cas1, Cas1 and IL-1β protein levels in DRG tissues of AAV-*Ppm1a*-*shRNA*-injected PIPN mice (*n* = 3 per group). All values are presented as mean ± SEM. Statistical significance was determined using one-way ANOVA followed by Dunnett’s *post hoc* test. Exact P values are shown above the bars

Immunofluorescence assay results revealed elevated macrophage accumulation (labeled by CD68, green) in the DRG of PIPN mice, which was attenuated by OB treatment (2.5 and 5 mg/kg) (Fig. 4a). Consistently, qPCR results further demonstrated the upregulation of M1-associated genes (*Tnfα*, *Il6* and *Cd86*) and the downregulation of M2 markers (*Arg1*, *Il10* and *Cd206*) in the DRG of PIPN mice(*P* < 0.001, *P* < 0.001, *P* = 0.004, *P* < 0.001, *P* = 0.006, *P* < 0.001, respectively), both of which were reversed by OB treatment (*P* < 0.001, *P* = 0.002, *P* = 0.002, *P* < 0.001, *P* = 0.009, *P* < 0.001, respectively; Fig. 4b and c). However, these effects were abolished in the PIPN mice injected with AAV-*Ppm1a*-*shRNA*, indicating that OB regulates macrophage polarization by targeting PPM1A (*P* = 0.06, *P* > 0.99, *P* = 0.12, *P* = 0.60, *P* = 0.95, *P* = 0.99, respectively; Fig. 4d and e).

#### OB suppresses NLRP3 inflammasome-mediated M1 polarization of macrophages in PIPN mice by targeting PPM1A

Given the critical role of NLRP3 inflammasome activation in promoting M1 polarization and pro-inflammatory cytokine release [42], we assessed the expression levels of inflammasome-related genes and proteins in DRG tissues of PIPN mice.

qPCR results indicated that OB treatment antagonized the elevation of mRNA levels of *Il1β* and *Nlrp3* (*P* < 0.001 for all; Fig. 4f and g), while immunofluorescence analysis showed that OB treatment inhibited the co-localization of NLRP3 and ASC (inflammasome assembly) (Fig. 4h). Similarly, western blot results demonstrated that OB repressed the protein levels of NLRP3, cleaved-Caspase1 (cleaved-Cas1) and IL-1β (*P* = 0.01, *P* = 0.02, *P* = 0.005, respectively; Fig. 4i and j). Notably, the above effects of OB were all abolished in the DRG tissues of the AAV-*Ppm1a*-*shRNA*-injected PIPN mice, as demonstrated in qPCR (*P* = 0.88, *P* = 0.78, respectively; Fig. 4k and l), immunofluorescence (Fig. 4m) and western blot (*P* = 0.44, *P* = 0.14, *P* = 0.49, respectively; Fig. 4n and o) results.

#### OB reverses PTX/ATP-induced M1 polarization and inflammasome activation in BMDMs

**Fig. 5:**
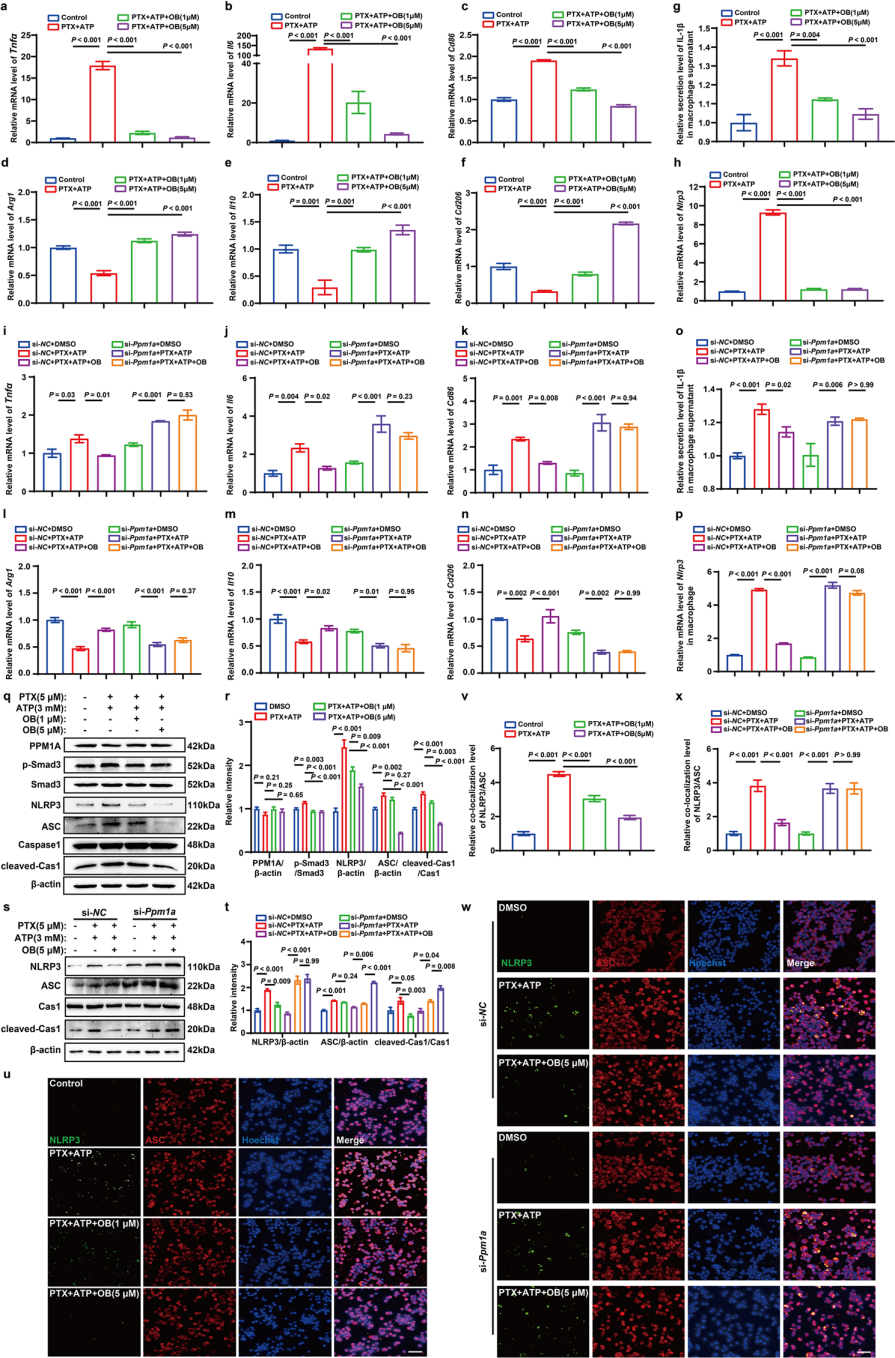
OB suppresses M1 polarization of macrophages via PPM1A *in vitro*. Macrophages were pretreated with OB (1 or 5 µM) for 24 h, followed by stimulation with PTX (5 µM) for 3.5 h and ATP (3 mM) for 0.5 h. qPCR analysis of **(a-c)** M1 (*Tnfα*, *Il6* and *Cd86*) and **(d-f)** M2 macrophage markers (*Arg1*, *Il10* and *Cd206*) mRNA levels in macrophages (*n* = 3 per group). **g** ELISA measurement of IL-1β concentration in the supernatant of macrophages (*n* = 3 per group). **h** qPCR analysis of *Nlrp3* mRNA levels in macrophages (*n* = 3 per group). Macrophages were transfected with si-*NC* or si-*Ppm1a* and treated with OB (5 µM), followed by stimulation with or without PTX/ATP. qPCR analysis of M1 **(i**, *Tnfα*; **j**, *Il6*; **k**, *Cd86***)** and M2 **(l**, *Arg1*; **m**, *Il10*; **n**, *Cd206***)** markers in siRNA-transfected macrophages (*n* = 3 per group). **(o)** ELISA measurement of IL-1β concentration in the supernatant of macrophages transfected with siRNA (*n* = 3 per group). **p** qPCR analysis of *Nlrp3* mRNA level in macrophages transfected with siRNA (*n* = 3 per group). **q**,** r** Western blot analysis and quantification of PPM1A, p-Smad3, Smad3, NLRP3, ASC, cleaved-Cas1 and Cas1 protein levels in macrophages (*n* = 3 per group). **s**,** t** Western blot analysis and quantification of NLRP3, ASC, cleaved-Cas1 and Cas1 protein levels in macrophages transfected with siRNA (*n* = 3 per group). **u**,** v** Representative immunofluorescence images with quantification of NLRP3 (green) and ASC (red) in macrophages (*n* = 5 per group). Scale bar: 50 μm. **w**,** x** Representative immunofluorescence images with quantification of NLRP3 (green) and ASC (red) in macrophages transfected with siRNA (*n* = 5 per group). Scale bar: 50 μm. All values are presented as mean ± SEM. Statistical significance was determined using one-way ANOVA followed by Dunnett’s *post hoc* test. Exact P values are shown above the bars

Additionally, we validated the role of PPM1A in OB-mediated regulation of inflammation in BMDMs stimulated by PTX and ATP, as described previously [43, 44]. qPCR results showed that PTX/ATP stimulation increased the mRNA levels of M1 macrophage markers (*Tnfα*, *Il6* and *Cd86*) (*P* < 0.001 for all) and suppressed M2 markers (*Arg1*, *Il10* and *Cd206*) (*P* < 0.001, *P* = 0.001, *P* < 0.001, respectively) in BMDMs. OB treatment reversed these transcriptional changes (*P* < 0.001 for all; Fig. 5a-f) without affecting cell viability (*P* > 0.99 for all; Fig. S5a). Consistently, ELISA results demonstrated that OB reduced IL-1β secretion (*P* < 0.001; Fig. 5g). Since IL-1β secretion in macrophages is associated with NLRP3 inflammasome activation [45, 46], we further investigated whether OB regulates inflammasome activation. qPCR results showed that OB suppressed the PTX/ATP-induced upregulation of *Nlrp3* mRNA levels (*P* < 0.001 for all; Fig. 5h), indicating that OB inhibits both NLRP3 inflammasome activation and M1 polarization.

#### OB inhibits PTX/ATP-induced inflammation by targeting PPM1A in BMDMs

To determine whether OB inhibits M1 macrophage polarization by targeting PPM1A, we performed siRNA-mediated PPM1A knockdown in BMDMs. The knockdown efficacy of PPM1A was confirmed by western blot assay (*P* < 0.001; Fig. S5b and c). In PPM1A-knockdown BMDMs, OB no longer suppressed the expression of M1 markers (*Tnfα*, *Il6* and *Cd86*) (*P* = 0.53, *P* = 0.23, *P* = 0.94, respectively; Fig. 5i-k) nor restored the levels of M2 markers (*Arg1*, *Il10*, *Cd206*) (*P* = 0.37, *P* = 0.95, *P* > 0.99, respectively; Fig. 5l-n). Similarly, ELISA results indicated that OB had no effect on IL-1β secretion in PPM1A-knockdown BMDMs (*P* > 0.99; Fig. 5o). Consistent with these findings, qPCR results showed that OB did not reduce *Nlrp3* mRNA levels in PPM1A-knockdown BMDMs (*P* = 0.08; Fig. 5p).

Western blot results demonstrated that OB inhibited PTX/ATP-induced upregulation of NLRP3 and cleaved-Cas1 (*P* < 0.001 for all; Fig. 5q and r), but this effect was abolished in PPM1A-knockdown BMDMs (*P* = 0.99, *P* = 0.008, respectively; Fig. 5s and t). Similarly, immunofluorescence results revealed that OB disrupted NLRP3-ASC co-localization in control BMDMs, but not in those transfected with si-*Ppm1a* (Fig. 5u-x).

Collectively, all results demonstrate that OB suppresses M1 macrophage polarization and NLRP3 inflammasome activation by targeting PPM1A.

### OB targets PPM1A to suppress neuroinflammation in macrophages by inhibiting IKK/NF-κB pathway

#### OB suppresses neuroinflammation in macrophages by inhibiting IKK/NF-κB pathway

**Fig. 6:**
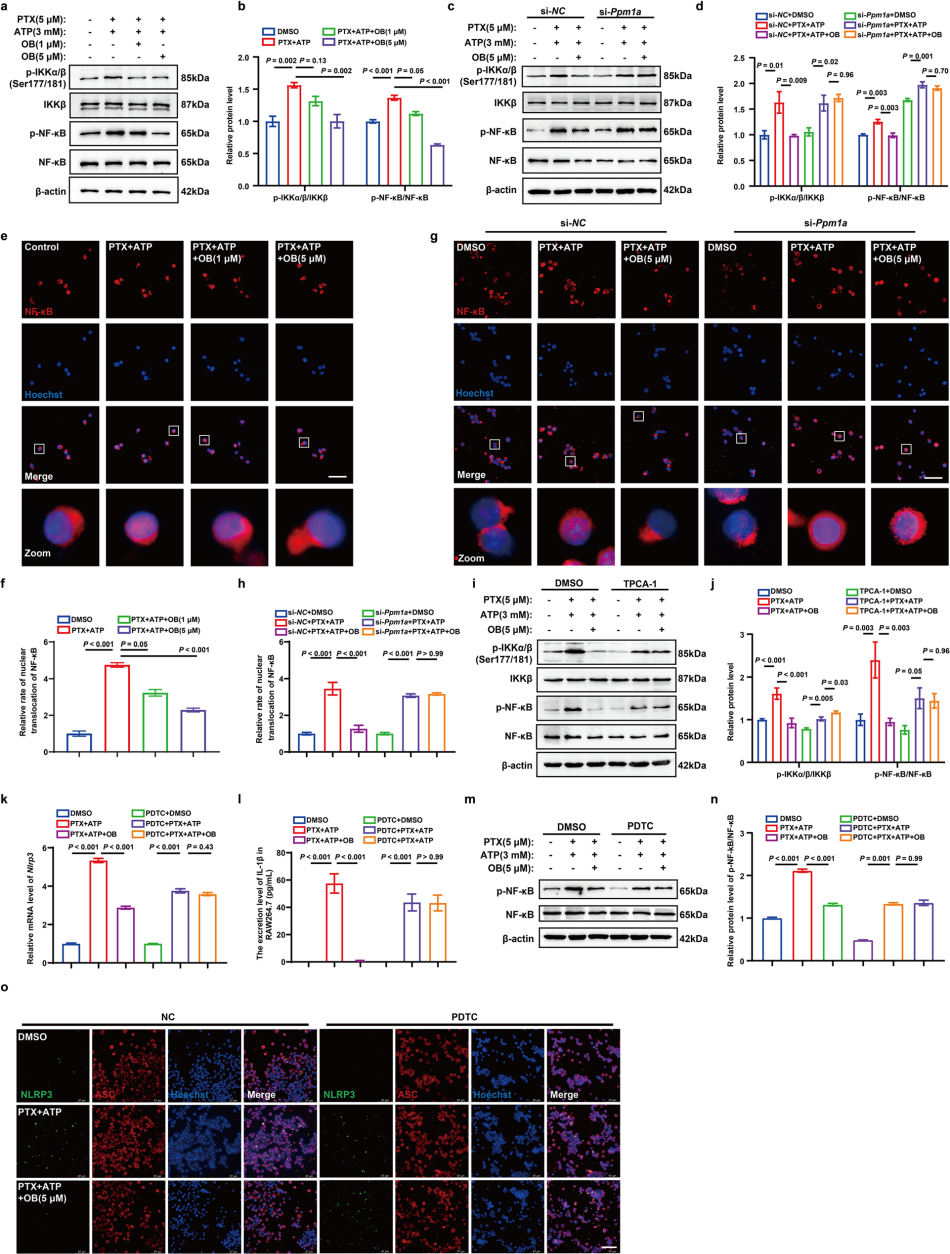
OB suppresses neuroinflammation in macrophages by inhibiting NF-κB nuclear translocation. Macrophages were pretreated with OB (1 or 5 µM) for 24 h, followed by treatment with PTX (5 µM) and OB for 3.5 h, and then stimulated with ATP (3 mM) for 0.5 h. **a**,** b** Western blot analysis and quantification of p-IKKα/β(Ser177/181), IKKβ, p-NF-κB and NF-κB protein levels in macrophages (*n* = 3 per group). **c**,** d** Western blot and quantification of p-IKKα/β(Ser177/181), IKKβ, p-NF-κB and NF-κB protein levels in macrophages transfected with siRNA (*n* = 3 per group). **e**,** f **Representative immunofluorescence images with quantification of NF-κB (red) nuclear translocation in macrophages (*n* = 5 per group). Scale bar: 50 μm. **g**,** h** Representative immunofluorescence images with quantification of NF-κB (red) nuclear translocation in macrophages transfected with siRNA (*n* = 5 per group). Scale bar: 50 μm. Macrophages were pretreated with OB (5 µM) for 24 h and then with TPCA-1 (5 µM) for 1 h, followed by stimulation with PTX (5 µM) for 3.5 h and ATP (3 mM) for 0.5 h. **i**,** j** Western blot analysis and quantification of p-IKKα/β(Ser177/181), IKKβ, p-NF-κB and NF-κB protein levels in macrophages pretreated with an IKKβ inhibitor TPCA-1 (*n* = 3 per group). Macrophages were pre-incubated with OB for 24 h, then treated with PDTC for 1 h, followed by stimulation with PTX for 3.5 h and subsequently incubated with ATP for 0.5 h. **k** qPCR analysis of *Nlrp3* mRNA level in macrophages treated with PDTC (*n* = 3 per group). **l** ELISA detection of IL-1β levels in the supernatant of macrophages treated with PDTC (*n* = 3 per group). **m**,** n** Western blot and quantification of p-NF-κB and NF-κB protein levels in macrophages treated with PDTC (*n* = 3 per group). **o** Representative immunofluorescence images of NLRP3 (green) and ASC (red) in macrophages treated with PDTC (*n* = 5 per group). Scale bar: 67 μm. All values are presented as mean ± SEM. Statistical significance was determined using one-way ANOVA followed by Dunnett’s *post hoc* test. Exact P values are shown above the bars

The transcription of NLRP3 is tightly regulated by the nuclear factor kappa B (NF-κB) signaling pathway [47–49]. Since NF-κB requires the phosphorylation of its inhibitory subunit IκBα by the IκB kinase (IKK) complex with IKKβ serving as the key kinase responsible for this event [50], we investigated whether OB suppresses neuroinflammation in macrophages via inhibition of the IKK/NF-κB axis. Western blot results showed that OB treatment reduced the levels of phosphorylated Ikkα/β (p-Ikkα/β) and phosphorylated NF-κB (p-NF-κB) in PTX/ATP-stimulated BMDMs (*P* = 0.002 for all, respectively; Fig. 6a and b), while these inhibitory effects were counteracted in PPM1A-knockdown BMDMs (*P* = 0.96, *P* = 0.70, respectively; Fig. 6c and d).

Immunofluorescence assay results further confirmed that OB suppressed the nuclear translocation of NF-κB following PTX/ATP stimulation in BMDMs (*P* < 0.001 for all; Fig. 6e and f). However, this suppression was not observed in PPM1A-knockdown BMDMs (*P* > 0.99; Fig. 6g and h), indicating that PPM1A is required for OB-induced inhibition of NF-κB nuclear translocation. To further demonstrate the involvement of IKK in this process, BMDMs were treated with TPCA-1, a selective IKKβ inhibitor. As shown in Fig. 6i and j, TPCA-1 treatment markedly reduced PTX/ATP-induced phosphorylation of IKKα/β and NF-κB. Notably, combined treatment with OB and TPCA-1 did not produce additional inhibitory effects beyond TPCA-1 alone (*P* = 0.03, *P* = 0.96, respectively; Fig. 6i and j), indicating that OB and TPCA-1 act through the same signaling axis and that the anti-inflammatory effect of OB is mediated via the IKK/NF-κB pathway. Additionally, co-IP assays performed in DRG macrophages indicated that PPM1A did not directly interact with NF-κB (Fig. S6a). This suggests that the inhibition of NF-κB by PPM1A is not due to direct interaction, but is mediated through its action on the upstream kinase IKKβ.

To further assess the role of NF-κB in OB-induced inflammasome regulation, BMDMs were pretreated with NF-κB inhibitor pyrrolidine dithiocarbamate (PDTC) [51]. qPCR assay results revealed that PDTC abolished the OB-induced reduction of *Nlrp3* mRNA levels (*P* < 0.001, *P* = 0.43, respectively; Fig. 6k). Consistently, ELISA results indicated that PDTC also abolished the OB-induced decrease in IL-1β secretion (*P* < 0.001, *P* > 0.99, respectively; Fig. 6l). Furthermore, western blot analysis demonstrated that PDTC abolished the inhibitory effect of OB on PTX/ATP-induced upregulation of p-NF-κB protein levels (*P* < 0.001, *P* = 0.99, respectively; Fig. 6m and n). Similarly, immunofluorescence assay results showed that PDTC pre-treatment abolished the ability of OB to reduce NLRP3 and ASC co-localization in BMDMs (Fig. 6o).

#### OB targets PPM1A to suppress neuroinflammation in macrophages

To further determine whether the anti-neuroinflammatory effects of OB are mediated through its known antispasmodic targets, we performed siRNA-mediated knockdown of *Chrm3* (muscarinic M3 receptor), *Tacr2* (tachykinin NK2 receptor), and *Cacna1c* (L-type calcium channel) in macrophages. Crucially, OB retained its ability to suppress PTX-induced NF-κB activation and pro-inflammatory cytokine release despite the deficiency of these individual canonical targets (*P* < 0.001, *P* = 0.01, *P* = 0.006, *P* = 0.03, *P* < 0.001, *P* < 0.001, *P* = 0.008, *P* = 0.01, *P* < 0.001, *P* < 0.001, *P* = 0.01, *P* = 0.003, respectively; Fig. S7a–l). These results indicate that the efficacy of OB in PIPN is not primarily dependent on its traditional gastrointestinal-related pathways, supporting PPM1A as its predominant functional target in this context.

These findings demonstrate that OB suppresses neuroinflammation in macrophages by inhibiting NF-κB signaling via targeting PPM1A.

### OB inhibits macrophage-neuron crosstalk by targeting PPM1A

Given the ability of OB to alleviate macrophage-mediated inflammation and neuronal damage in DRG, we further explored its regulatory effect on the bidirectional interaction between macrophages and neurons.

#### OB protects DRG neurons from macrophage-induced damage via PPM1A

**Fig. 7:**
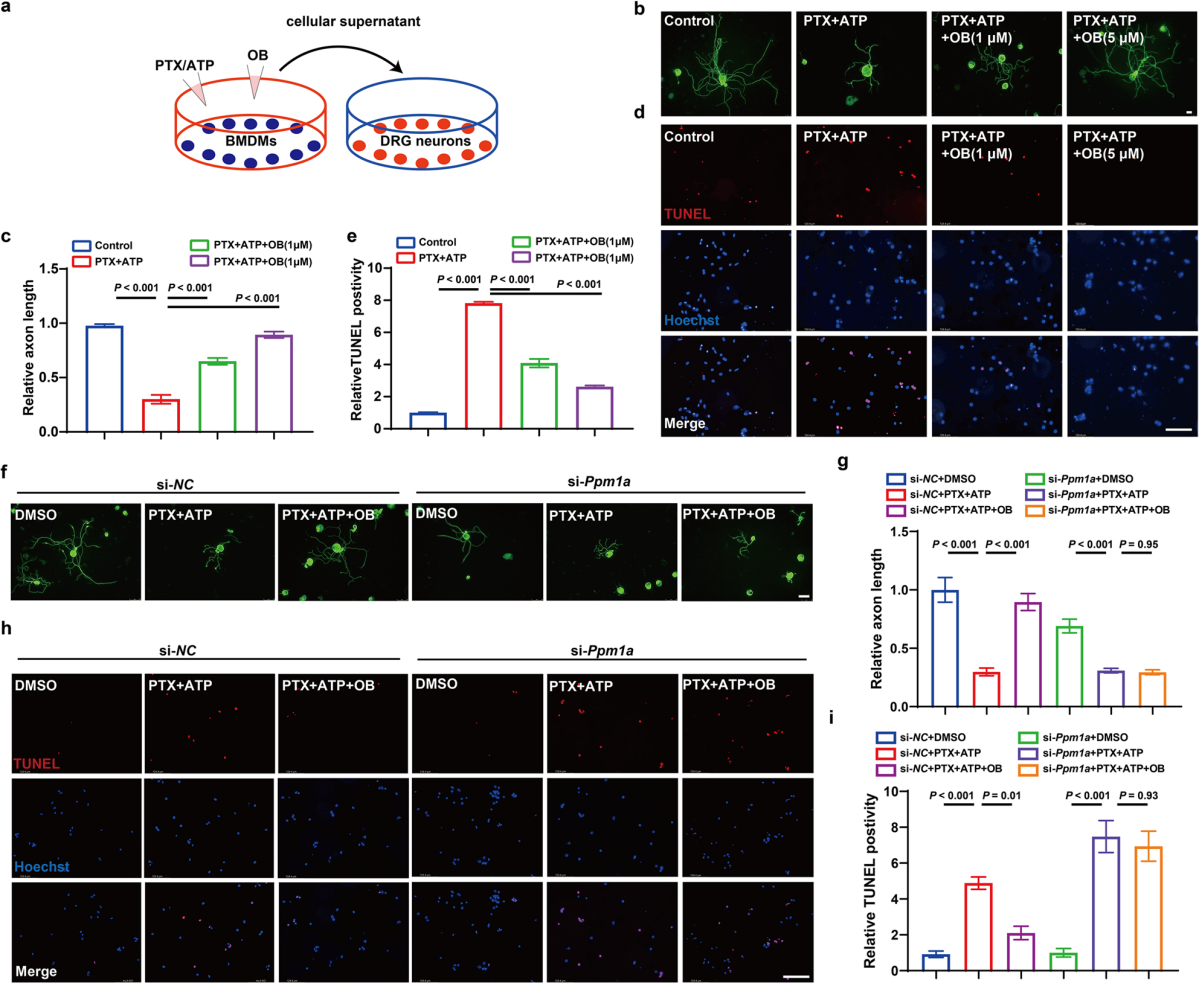
OB protects DRG neurons from BMDM-conditioned medium-induced neurite damage and apoptosis.** a** BMDMs were pretreated with OB (1 and 5 µM) for 24 h, followed by stimulation with PTX (5 µM) for 3.5 h and ATP (3 mM) for 0.5 h. DRG neurons were incubated with above mentioned BMDMs conditioned medium (CM) for 24 h. **b**,** c** Representative immunofluorescence images with quantification of total neurite length in primary DRG neurons (labeled by β-tubulin III, green) (*n* = 4 per group). Scale bars: 25 μm. **d**,** e** TUNEL assay detecting the cell apoptosis of primary DRG neurons (*n* = 4 per group). Scale bar: 124.4 μm. **f**,** g** Representative immunofluorescence images with quantification of total neurite length of primary DRG neurons transfected with siRNA (*n* = 4 per group). Scale bars:50 μm. **h**,** i **TUNEL assay detecting the cell apoptosis of primary DRG neurons transfected with siRNA (*n* = 4 per group). Scale bar: 124.4 μm. All values are presented as mean ± SEM. Statistical significance was determined using one-way ANOVA followed by Dunnett’s *post hoc* test. Exact P values are shown above the bars

To assess the influence of OB on DRG neuronal injury induced by inflammatory macrophages, a conditioned medium assay was performed. As illustrated in Fig. 7a, BMDMs were pretreated with OB (1, 5 µM) for 24 h, followed by stimulation with PTX (5 µM) for 3.5 h and ATP (3 mM) for 30 min. After stimulation, the medium was replaced to remove stimulants, and the conditioned medium (CM) was collected after an additional 24 h and applied to culture primary DRG neurons for 24 h. Immunofluorescence and TUNEL assays revealed that CM derived from PTX/ATP-stimulated BMDMs impaired neurite outgrowth (*P* < 0.001 for all; Fig. 7b and c) and increased neuronal apoptosis (*P* < 0.001 for all; Fig. 7d and e), whereas OB treatment attenuated these effects (*P* < 0.001 for all; Fig. 7b-e). Notably, the protective effect of OB was abolished when neurons were treated with CM from PPM1A-knockdown BMDMs, as indicated by reduced neurite outgrowth (*P* = 0.95; Fig. 7f and g) and increased apoptosis (*P* = 0.93; Fig. 7h and i). To validate the critical role of PPM1A in PIPN, additional rescue experiments were conducted. In primary DRG neurons, overexpression of PPM1A via plasmid transfection effectively reversed PTX-mediated suppression of neurite outgrowth (*P* < 0.001; Fig. S8a and b). These results highlighted the essential role of PPM1A in mediating the neuroprotection of OB.

#### OB attenuates macrophage activation in response to damaged neuron-derived signals

**Fig. 8:**
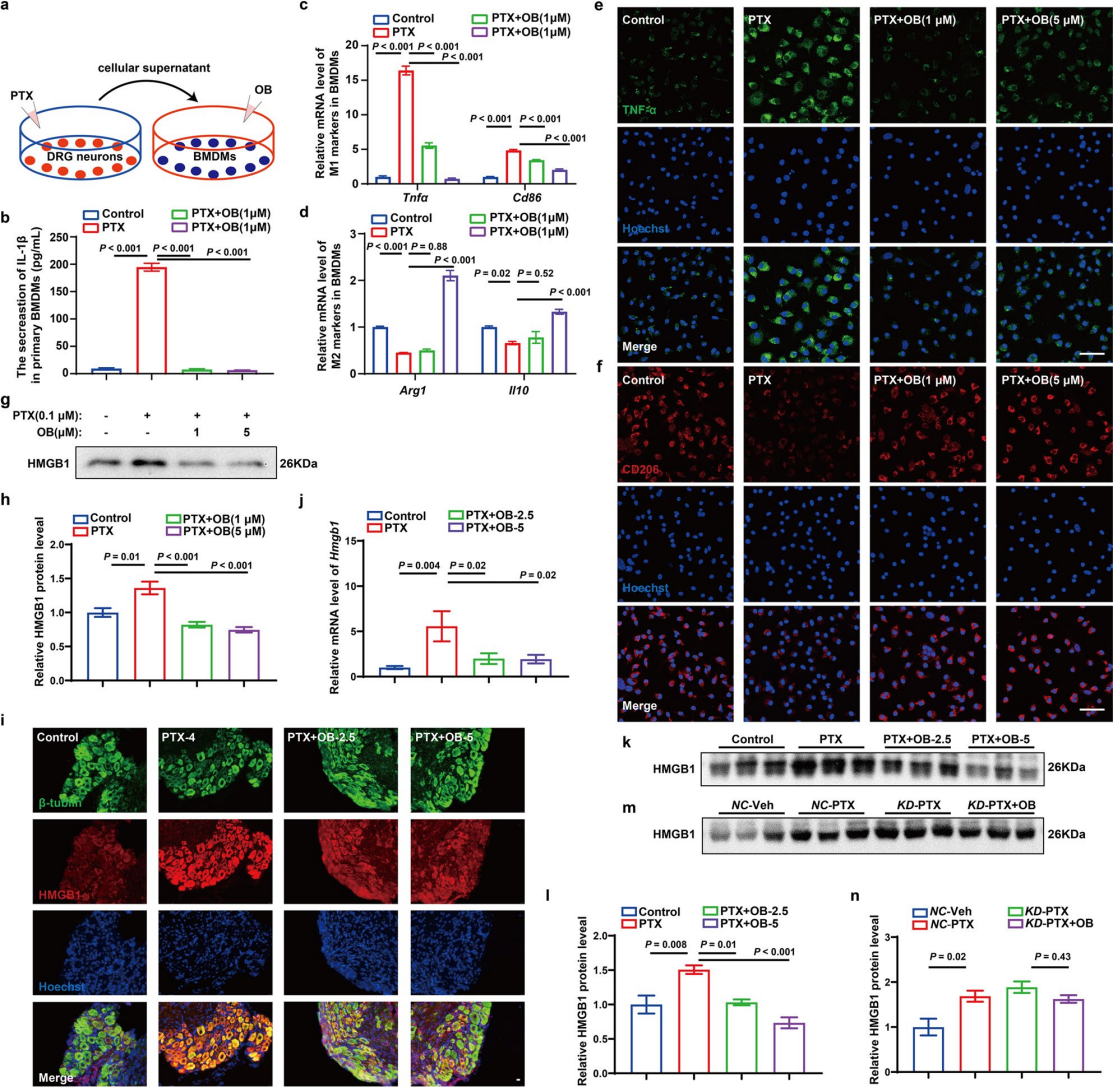
OB suppresses macrophage M1 polarization induced by PTX-mediated HMGB1 release from DRG neurons.** a** Primary DRG neurons were pretreated with OB (1 and 5 µM) for 24 h, followed by PTX (0.1 µM) treatment for 24 h. BMDMs were incubated with the above mentioned DRG neuron CM for 24 h. **b** ELISA assay detecting the concentration of IL-1β in CM. qPCR analysis of mRNA levels of **(c)** M1 (*Tnfα* and *Cd86*) and **(d)** M2 macrophage markers (*Arg1* and *Il10*) (*n* = 3 per group). Representative immunofluorescence images of **(e)** M1 (labeled by TNF-α, green) and **(f)** M2 macrophages (labeled by CD206, red) (*n* = 4 per group). Scale bar: 50 μm. **g**,** h **Western blot analysis and quantification of HMGB1 protein levels in the supernatant of PTX-treated primary DRG neurons (*n* = 3 per group). **i** Representative immunofluorescence images of HMGB1 (red) in DRG tissues (DRG neurons labeled by β-tubulin III, green) of PIPN mice (*n* = 4 per group). Scale bars: 10 μm. **j** qPCR analysis of *Hmgb1* mRNA levels in DRG tissues of PIPN mice (*n* = 8 per group). **k**,** l** Western blot analysis and quantification of HMGB1 protein levels in serum of PIPN mice (*n* = 3 per group). **m**,** n** Western blot analysis and quantification of serum HMGB1 protein levels in AAV-*Ppm1a*-*shRNA*-injected PIPN mice (*n* = 3 per group). All values are presented as mean ± SEM. Statistical significance was determined using one-way ANOVA followed by Dunnett’s *post hoc* test. ns, not significant; Exact P values are shown above the bars

To investigate whether damaged neurons may exacerbate macrophage-mediated neuroinflammation through a feedback mechanism, BMDMs were cultured with CM derived from PTX-treated DRG neurons (Fig. 8a). ELISA results revealed that the neuron-derived CM increased IL-1β secretion in BMDMs, while OB treatment reduced IL-1β secretion (*P* < 0.001 for all; Fig. 8b). Consistently, qPCR results showed that OB treatment downregulated M1 macrophage markers (*Tnfα* and *Cd86*) (*P* < 0.001 for all; Fig. 8c) and upregulated M2 macrophage markers (*Arg1* and *Il10*) (*P* < 0.001 for all; Fig. 8d). These findings were further confirmed by immunofluorescence staining, which indicated the decreased Cd86 level and increased CD206 level in macrophages exposed to CM derived from PTX-induced neuron damage (Fig. 8e and f).

Damaged neurons release cytokines that activate macrophages and exacerbate neuronal damage following peripheral axon injury [52]. High-mobility group box-1 (HMGB1) as a nuclear protein is released into the extracellular environment by necrotic or damaged cells [53]. In various nerve injury models, HMGB1 is confirmed to be elevated in both CNS and DRG neurons and promotes the activation of macrophages and the release of pro-inflammatory cytokines such as IL-1β [54–57]. As a pro-inflammatory factor, HMGB1 contributes to the induction and maintenance of hyperalgesia in neuropathic pain models [41, 57–59]. To investigate whether OB regulates this process, we examined HMGB1 levels in the supernatant of PTX-treated DRG neurons. Western blot results showed elevated HMGB1 secretion following PTX stimulation, which was reduced by OB treatment (*P* = 0.01, *P* < 0.001, respectively; Fig. 8g and h).

In addition, to determine whether OB exerts similar regulatory effects on HMGB1 levels *in vivo*, we assessed HMGB1 expression in the DRG and serum of PIPN mice.

Immunofluorescence assay revealed that the HMGB1 protein levels were elevated in DRG tissues of PIPN mice compared to those of control mice (Fig. 8i). Consistently, qPCR and western blot assays confirmed elevated *Hmgb1* mRNA level in DRG tissues (*P* = 0.004; Fig. 8j) and increased HMGB1 protein level in serum (*P* = 0.008; Fig. 8k and l). Notably, PPM1A knockdown abolished the inhibitory effect of OB on HMGB1 release in serum of PIPN mice (*P* = 0.43; Fig. 8m and n).

Together, these results indicate that OB modulates neuron-macrophage crosstalk by suppressing macrophage activation and HMGB1 release via PPM1A, thereby alleviating neuroinflammation and protecting DRG neurons from damage.

### OB has no impact on the antitumor effect of PTX

Given the involvement of PPM1A in various aspects of tumor progression [60], it is essential to evaluate whether OB, as a PPM1A activator, interferes with the anticancer efficacy of PTX. For this purpose, we assessed the impact of OB on PTX-mediated cytotoxicity using both* in vitro* and in vivo breast cancer models [61, 62].

MTT assays indicated that OB had no impact on cell viability and co-treatment with OB did not attenuate the antiproliferative effect of PTX in MCF-7 cells (*P* = 0.88, *P* = 0.99, respectively; Fig. S9a). Additionally, MCF-7 cells were subcutaneously implanted into NU/NU nude mice, and the tumor growth was monitored by fluorescence imaging at different stages (Day 0, 7 and 21) following the injection of a fluorescent substrate (Fig. S9b). The results demonstrated that PTX treatment (4 mg/kg) suppressed tumor progression (*P* = 0.01; Fig. S9c and d), and co-administration with OB did not impair this therapeutic effect (*P* = 0.86; Fig. S9c and d).

These results indicate that OB does not compromise the antitumor efficacy of PTX.

## Discussion

PIPN is a dose-limiting adverse effect of PTX treatment that currently lacks effective medication [2]. Although duloxetine suppresses pain and has been used in the clinical treatment of PIPN [63], its therapeutic efficacy is limited due to the presence of unbearable side effects such as anorexia, renal insufficiency, hepatic impairment and poor nerve repair [64, 65]. Therefore, it is essential to identify novel compounds and therapeutic targets for the treatment of PIPN.

PPM1A is involved in multiple cellular events, including stress response regulation and signal transduction, and its enzyme activity is crucial for maintaining cellular homeostasis [13]. In this study, we observed that PPM1A enzyme activity, rather than protein or mRNA levels, was significantly reduced in the DRG tissues of PIPN mice, implicating its functional impairment in the pathogenesis of PIPN. Similar patterns of functional PPM1A impairment have been observed in diabetic peripheral neuropathy, where reduced phosphatase activity leads to sustained activation of pro-inflammatory pathways [66]. Furthermore, we demonstrated that pharmacological activation of PPM1A by OB alleviated neuropathic pain in PIPN mice. These findings suggest that enhancing PPM1A enzyme activity may serve as a novel and promising therapeutic strategy for the treatment of PIPN.

PTX readily crosses the blood-nerve barrier and accumulates in DRG tissues, contributing to its toxic effects [67]. While neuronal damage has long been recognized as a central mechanism in PIPN, increasing evidence highlights the significant role of non-neuronal cells in its pathogenesis. Our previous research indicated that the activation of 5-HTR_2A_ in spinal astrocytes is crucial for the development of PIPN, and pharmacological inhibition of 5-HTR_2A_ alleviates PIPN [68]. In addition, PTX-induced macrophage infiltration into DRG tissues and upregulated pro-inflammatory cytokines such as TNF-α and IL-1β, exacerbating PIPN pathology [10]. In this study, we observed that PTX inhibited PPM1A enzyme activity in DRG macrophages, thereby removing its negative regulation of IKKβ/NF-κB signaling. Consistent with previous reports demonstrating that PPM1A acts as a specific phosphatase that terminates NF-κB activation by dephosphorylating IKKβ at Ser177/181, rather than directly targeting NF-κB [69], our findings demonstrated that the PTX-induced PPM1A activity suppression leads to sustained IKKβ phosphorylation and unrestrained NF-κB nuclear translocation. This process primes the NLRP3 inflammasome and promotes the release of pro-inflammatory cytokines such as IL-1β. By contrast, OB treatment suppressed the priming and assembly of the NLRP3 inflammasome in DRG macrophages and drove a phenotypic shift from the pro-inflammatory M1 toward the reparative M2 polarization. Given that M2 macrophages are essential for resolving inflammation and supporting axonal regeneration following nervous system injuries [70, 71], this immunomodulatory effect suggests that OB may provide more sustained efficacy than conventional anti-inflammatory agents that cause broad immunosuppression.

Following peripheral axon injury, damaged neurons release cytokines that induce the inflammatory activation of macrophages, thereby exacerbating neuronal damage [52]. HMGB1 is a widely expressed non-histone DNA-binding nuclear protein that can be released into the extracellular environment by various cells or passively released by necrotic or damaged cells [53]. Acting as an endogenous damage-associated molecular pattern, HMGB1 activates TLR4 signaling, triggering inflammatory processes [72, 73]. In various nerve injury models, HMGB1 levels were significantly elevated in the CNS and DRG neurons, where it binds to membrane receptors like TLR4 on macrophages and other immune cells, triggering their activation and the release of inflammatory cytokines such as IL-1β [54–57]. As a pro-inflammatory mediator, HMGB1 induces and sustains hyperalgesia in neuropathic pain models [41, 57–59]. We further demonstrated that PTX induces neuronal damage and the release of HMGB1 into the extracellular space. Extracellular HMGB1 in turn promotes M1 polarization of DRG macrophages and the production of inflammatory cytokines, which exacerbate neuronal injury. Importantly, OB disrupts this vicious cycle at both ends by limiting neuronal HMGB1 release and attenuating macrophage response to HMGB1. Furthermore, the consistency between DRG changes and serum HMGB1 levels suggests that circulating HMGB1 could serve as a non-invasive biomarker for monitoring PIPN severity and therapeutic response.

OB treatment attenuated peripheral nerve damage and preserved vascular integrity following neuroinflammation. Unlike conventional analgesics such as opioid receptor activators, OB treatment suppressed neuroinflammation, restored IENF density and recovered microvascular blood flow. CPP test further indicated that OB did not alter positional preference in PIPN mice (Fig. S4a and b), suggesting that its mechanism in improving PIPN pathology differs from that of traditional analgesics.

Unlike µ-opioid receptor agonists such as DALDA [28] that provide relief by blocking nociceptive transmission, OB acts as a disease-modifying agent via the PPM1A/NF-κB/NLRP3 axis. This pathological intervention effectively mitigates hypersensitivity while remaining devoid of the addictive or rewarding properties associated with opioids, offering a significant safety advantage for the long-term treatment of patients.

Additionally, as a quaternary ammonium compound characterized by low systemic absorption and limited blood-brain barrier penetration, OB exhibited minimal CNS toxicity [23, 25]. We also assessed CNS toxicity of OB (5 mg/kg, i.p.) using rotarod and open-field tests. The results showed that OB treatment had no significant impairment of motor coordination or spontaneous locomotor activity compared to vehicle-treated PIPN mice (Fig. S3). Furthermore, OB did not interfere with the antitumor efficacy of paclitaxel in xenograft mice. Collectively, these data suggest that OB is a safe and effective adjunct candidate for the prevention of PIPN during chemotherapy.

Despite these promising results, several limitations must be addressed. First, all experiments were conducted in male mice, leaving open the question of whether our results exhibit gender disparity and generalize to female subjects. Second, while systemic and regional knockdown approaches confirmed the functional necessity of PPM1A, cell-type-specific transgenic models (e.g., *LysM-Cre* or *Advillin-Cre*) are needed to precisely dissect the relative contributions of PPM1A in macrophages versus neurons.

Finally, although intraperitoneal administration of OB demonstrated efficacy in PIPN mice, its low oral bioavailability presents a translational hurdle. Consequently, the development of optimized delivery systems or novel formulations will be critical for progressing this strategy toward clinical application.

The translational potential of our findings warrants further exploration. Given that OB is already approved for clinical use as an antispasmodic agent, its repurposing for PIPN could be expedited. Future studies should focus on evaluation of the therapeutic window and optimal dosing regimen of OB, as well as its long-term safety profile with chronic administration.

In conclusion, we demonstrated that OB as a PPM1A activator effectively ameliorated the pathologies in PIPN mice. Mechanistically, PTX accumulation in the DRG tissues led to neuronal damage, which in turn triggered macrophage infiltration and their differentiation into the pro-inflammatory M1 phenotype, releasing inflammatory cytokines that further exacerbated neuronal damage. OB inhibited the M1 polarization of macrophages in the DRG of PIPN mice through the PPM1A/NF-κB/NLRP3 pathway, thereby reducing neuronal damage. Furthermore, our results indicated that OB could be co-administered with PTX without diminishing its antitumor efficacy. Our findings highlight the potential of PPM1A activation in ameliorating PIPN and suggest that OB could be a promising therapeutic agent for treating this side effect of PTX-based cancer chemotherapy. 

## Supplementary Information

Supplementary Material 1.

Supplementary Material 2.

## Data Availability

The data sets supporting the conclusions of this article are available from the corresponding author, on reasonable request.
