# Supplementary material for: Otilonium bromide ameliorates paclitaxel-induced peripheral neuropathy by targeting phosphatase PPM1A
Source: J Neuroinflammation. 2026 May 7;23:224. doi: 10.1186/s12974-026-03845-9 (PMC13321745; doi:10.1186/s12974-026-03845-9)

Fig. 1a

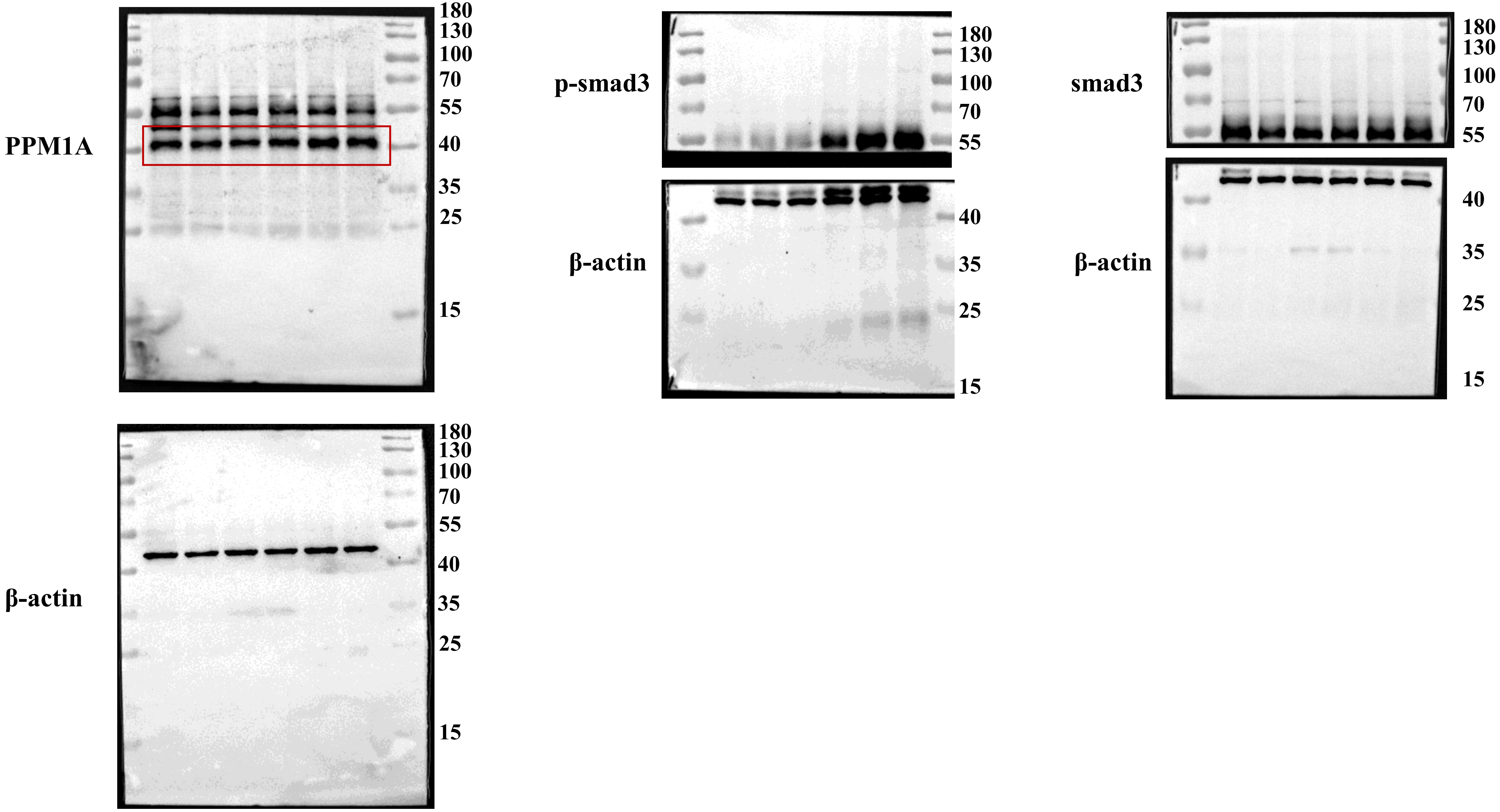

Fig. 4i

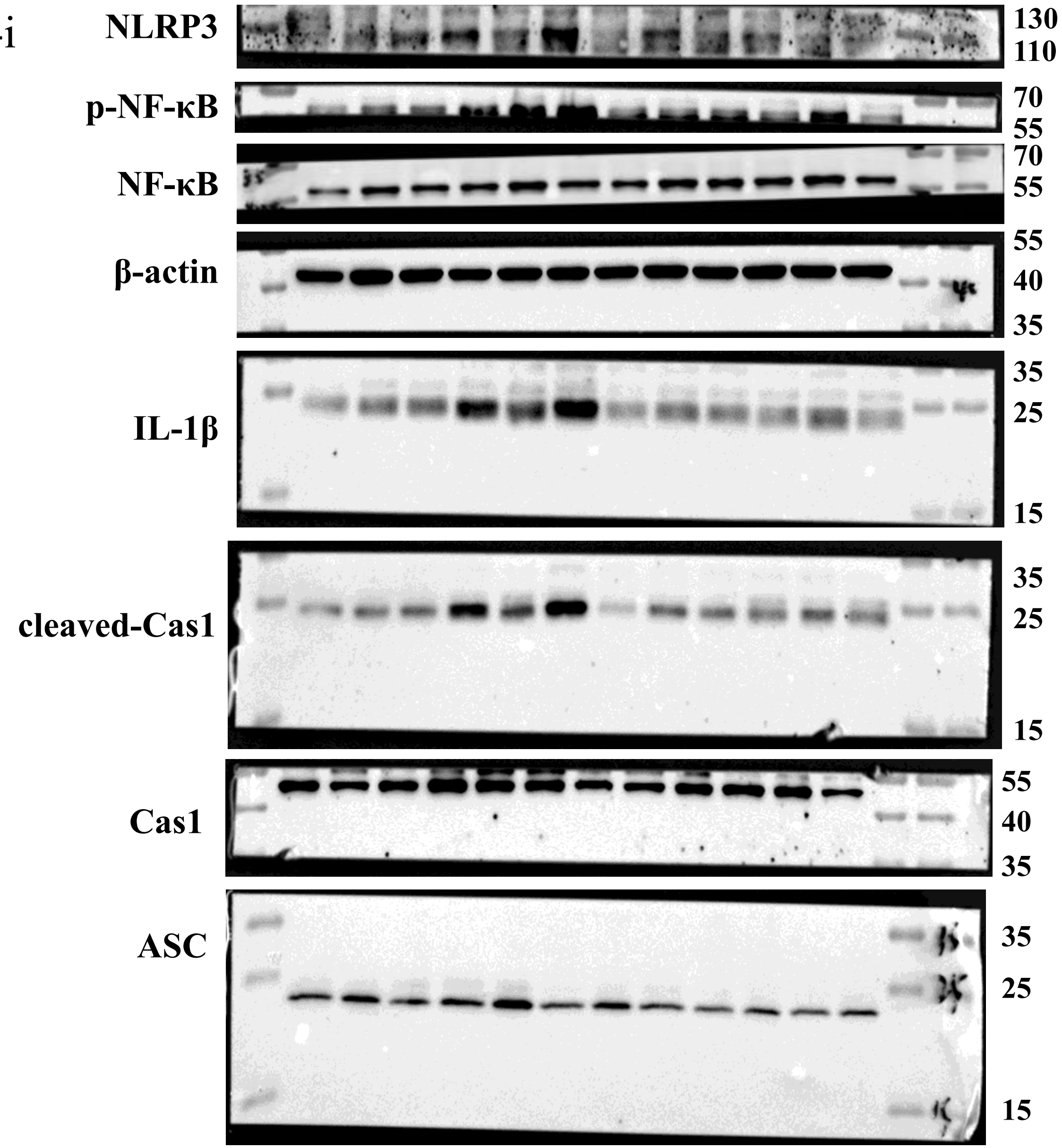
NLRP3
130
110
p-NF-κB
70
55
NF-κB
70
55
β-actin
55
40
35
IL-1β
35
25
15
cleaved-Cas1
35
25
15
Cas1
55
40
35
ASC
35
25
15

Fig. 4n

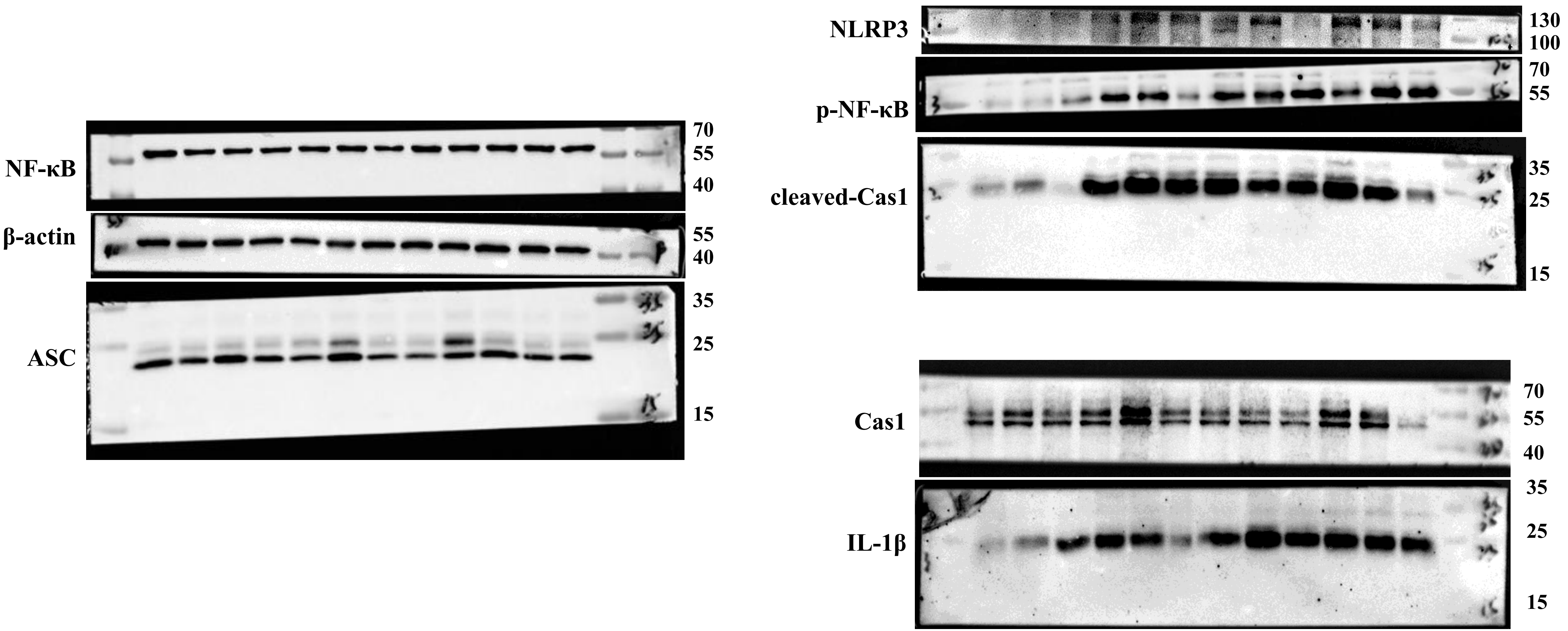

NLRP3
130
100
p-NF-κB
70
55
cleaved-Cas1
35
25
15
NF-κB
70
55
40
β-actin
55
40
ASC
35
25
15
Cas1
70
55
40
IL-1β
35
25
15

Fig. 5q

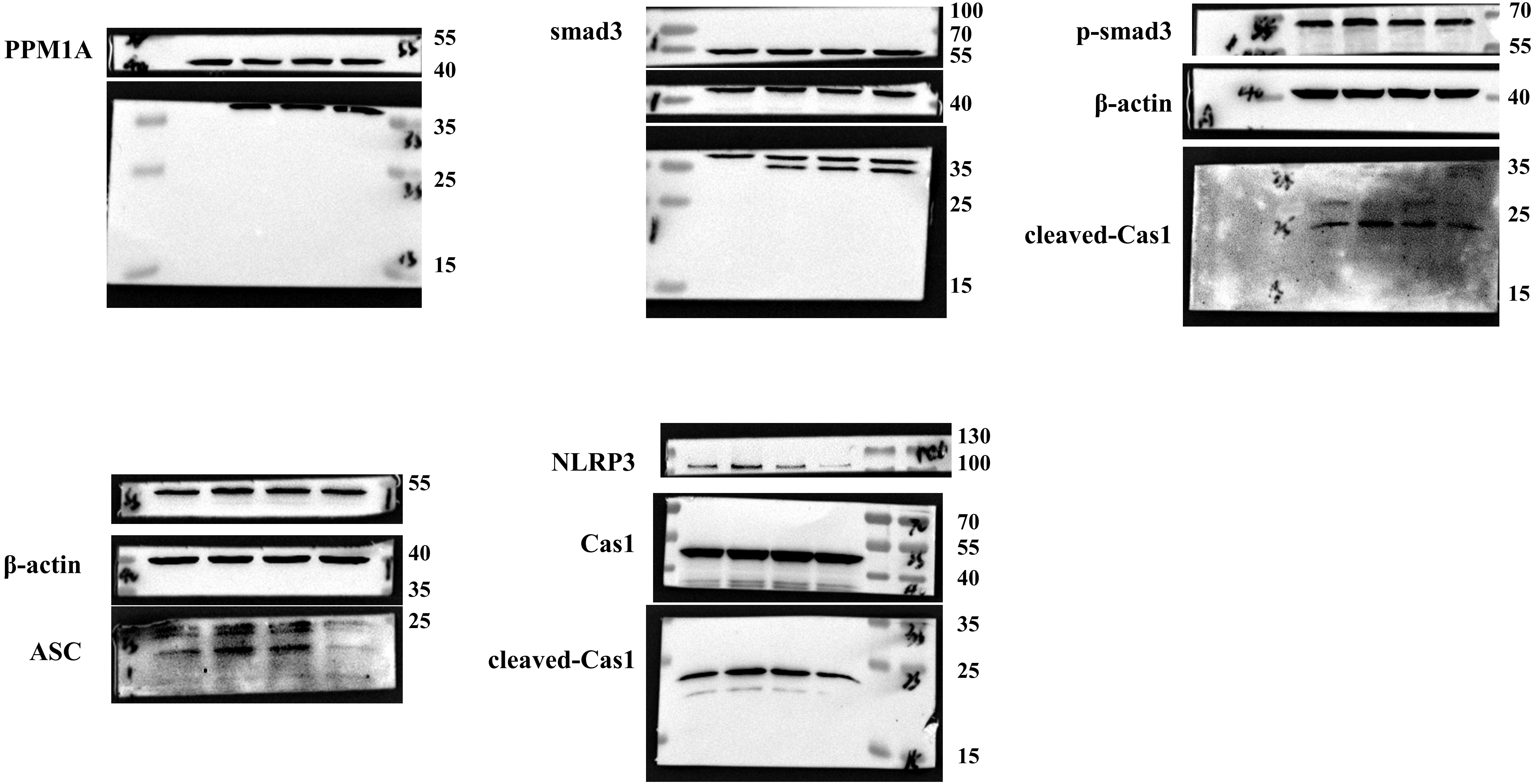
PPM1A
55
40
35
25
15
smad3
100
70
55
40
35
25
15
p-smad3
70
55
β-actin
40
cleaved-Cas1
35
25
15
55
β-actin
40
35
ASC
25
NLRP3
130
100
Cas1
70
55
40
cleaved-Cas1
35
25
15

Fig. 5s

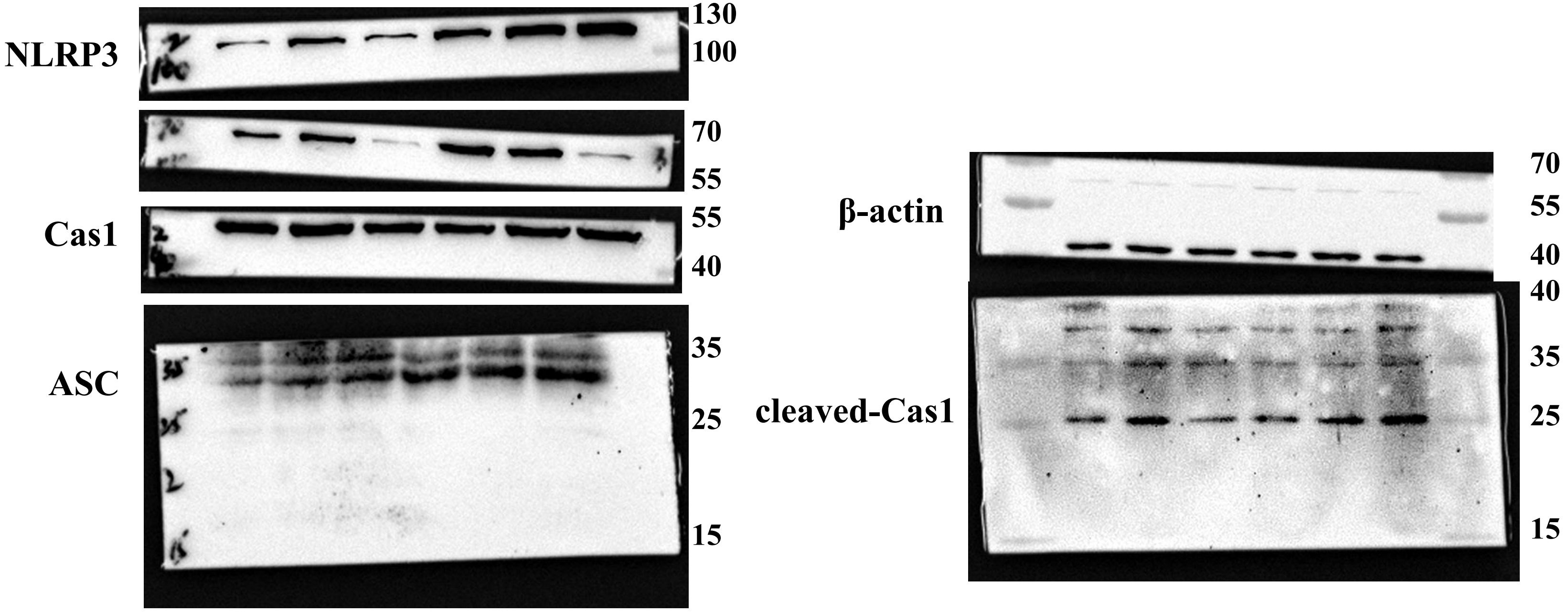
NLRP3
130
100
70
55
Cas1
55
40
ASC
35
25
15
β-actin
70
55
40
cleaved-Cas1
40
35
25
15

Fig. 6a

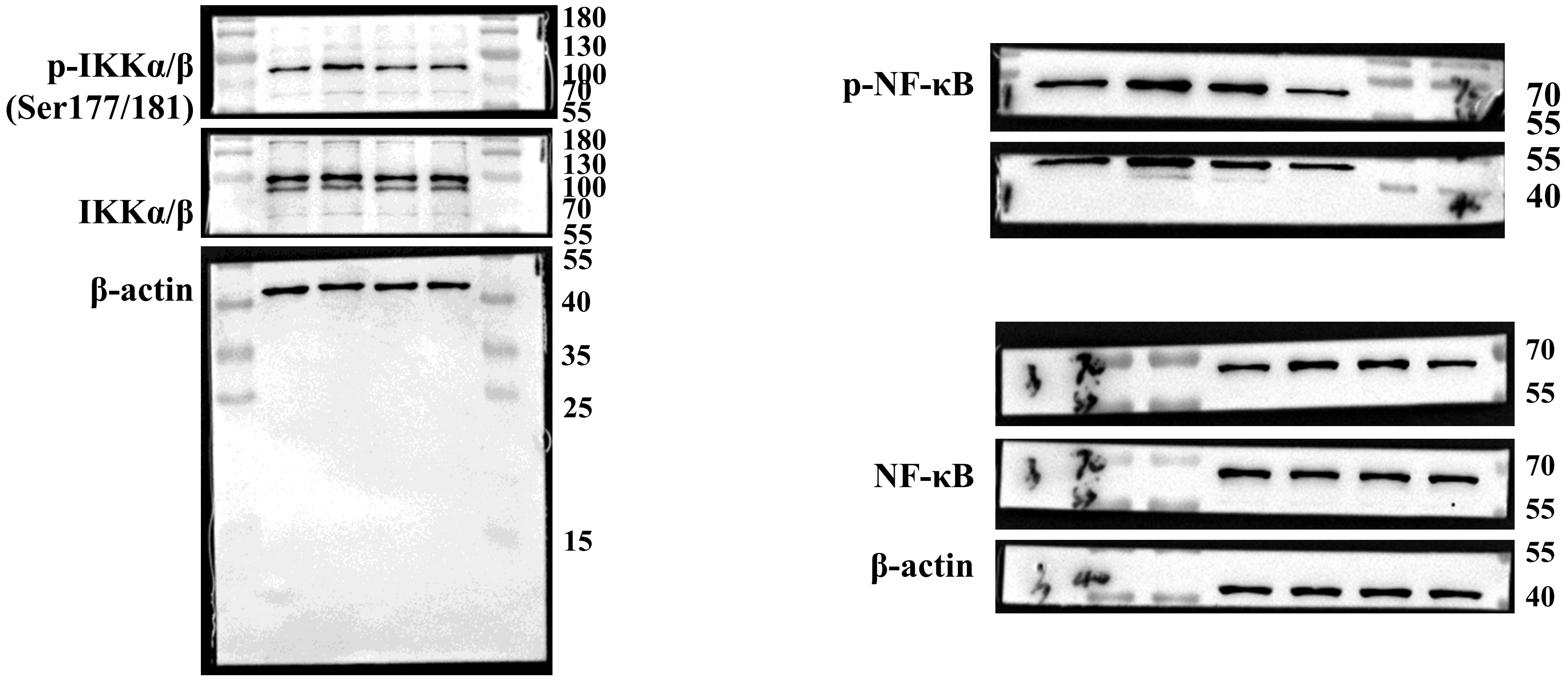
p-IKKα/β
(Ser177/181)
180
130
100
70
55
IKKα/β
180
130
100
70
55
β-actin
55
40
35
25
15
p-NF-κB
70
55
55
40
70
55
NF-κB
70
55
β-actin
55
40

Fig. 6c

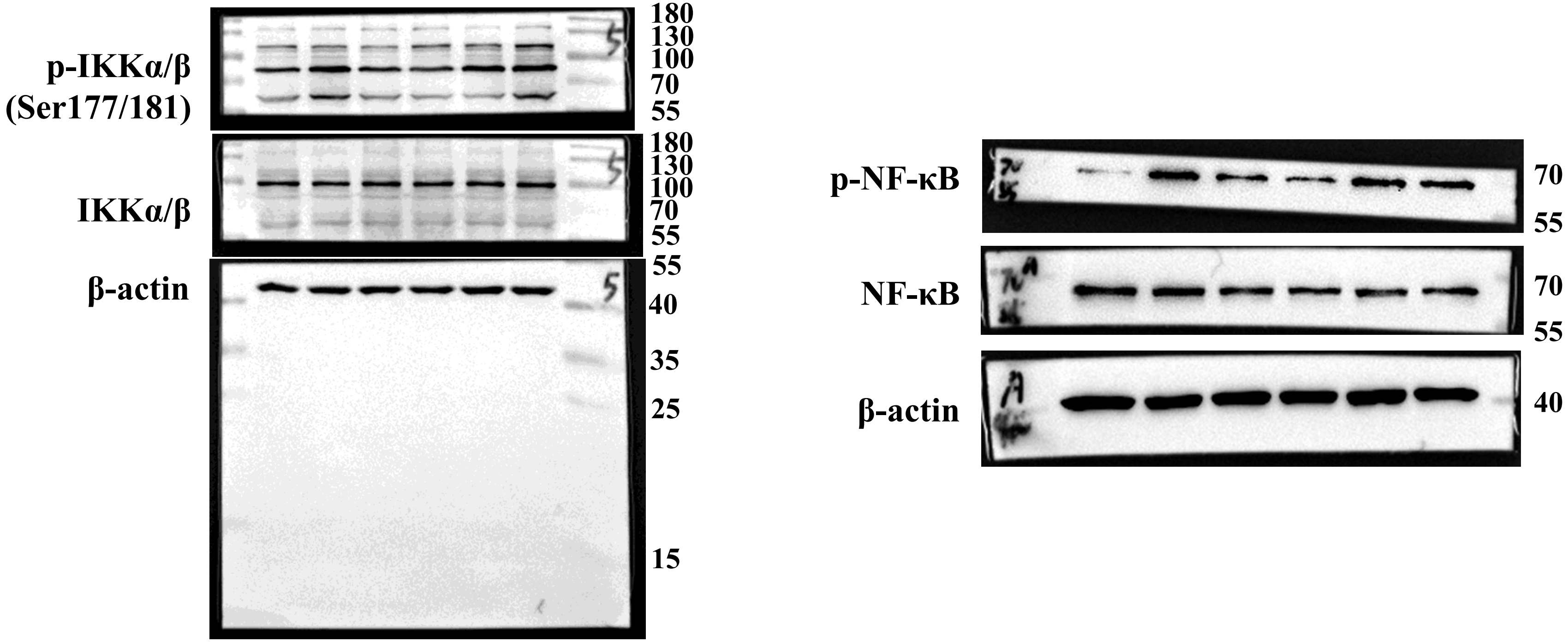
p-IKKα/β
(Ser177/181)
180
130
100
70
55
IKKα/β
180
130
100
70
55
β-actin
55
40
35
25
15
p-NF-κB
70
55
NF-κB
70
55
β-actin
40

Fig. 6i

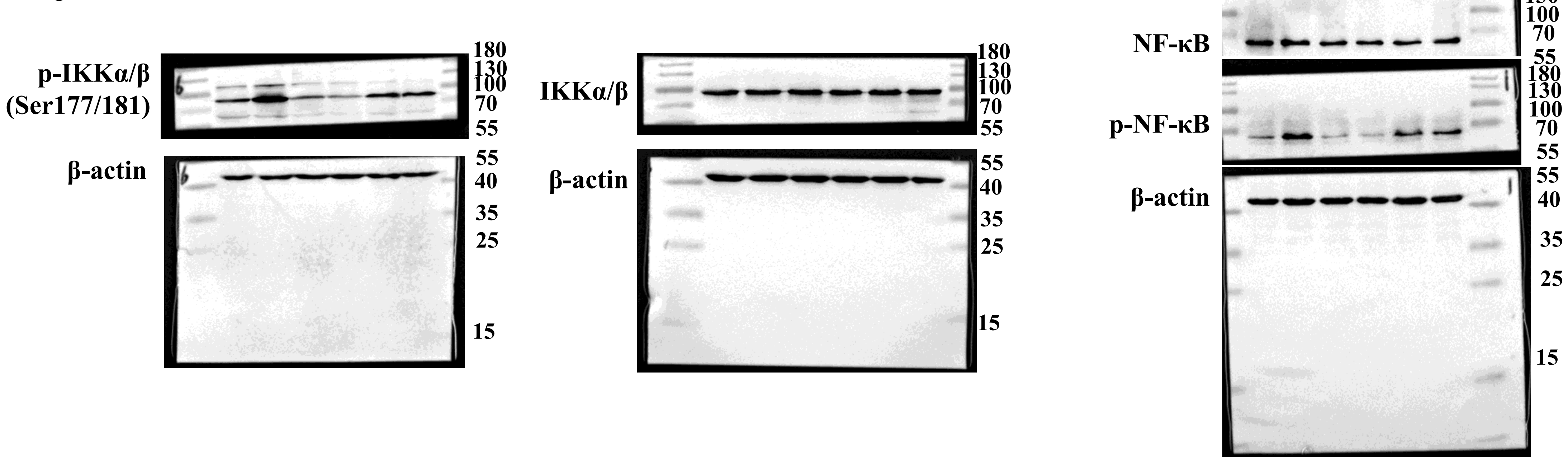
p-IKKα/β
(Ser177/181)
β-actin
IKKα/β
β-actin
NF-κB
p-NF-κB
β-actin
180
130
100
70
55
40
35
25
15

Fig. 6m

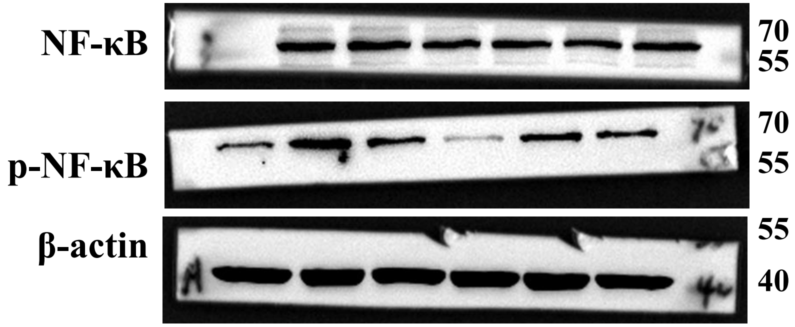
NF-κB
p-NF-κB
β-actin
70
55
40

Fig. 8g

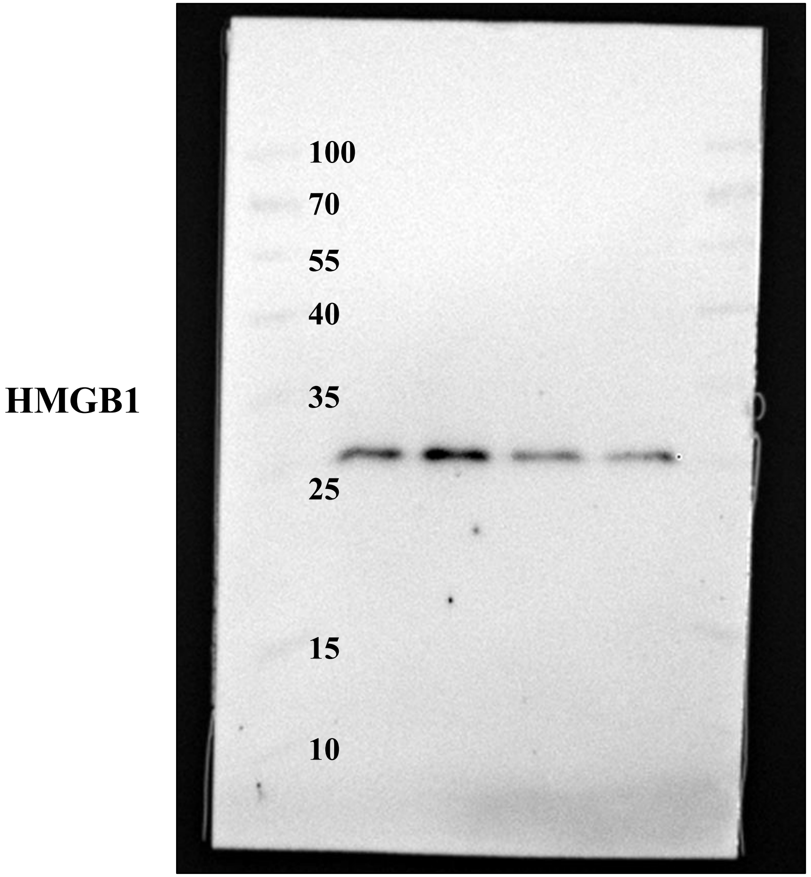

Fig. 8k

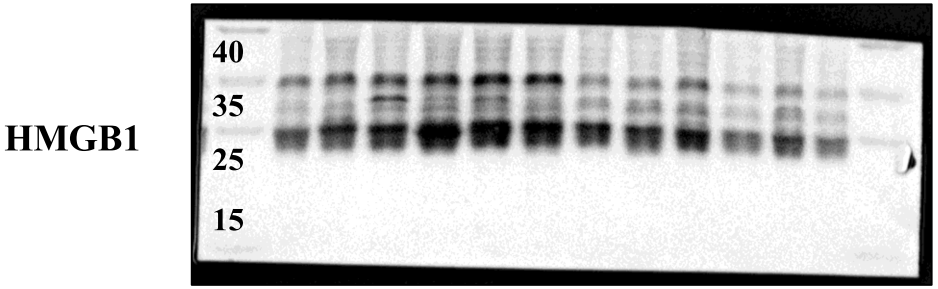

Fig8 m

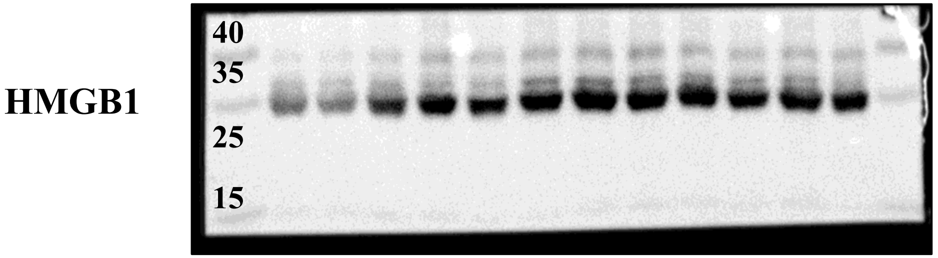

Fig. S2a

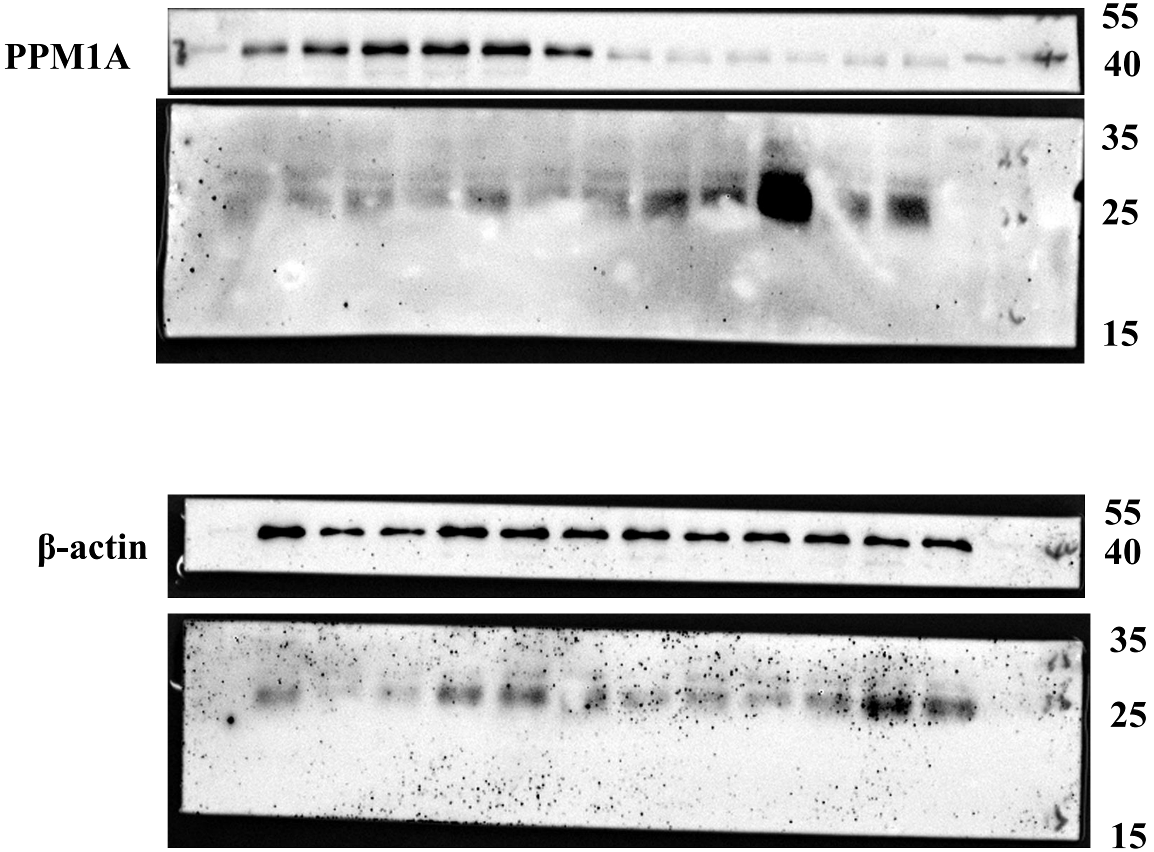

Fig. S5b

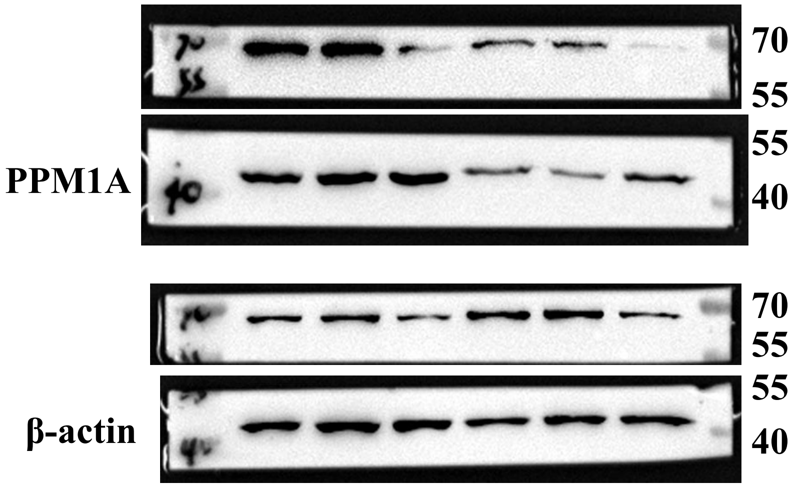

Fig. S6a

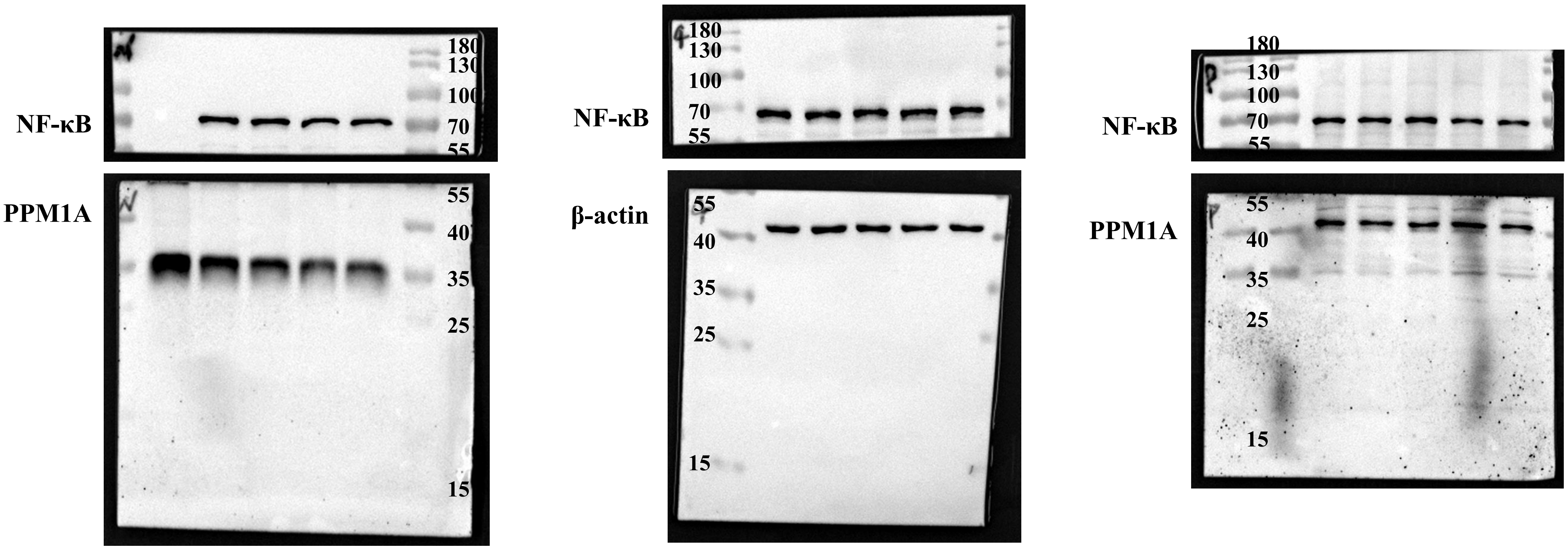

Fig. S7c

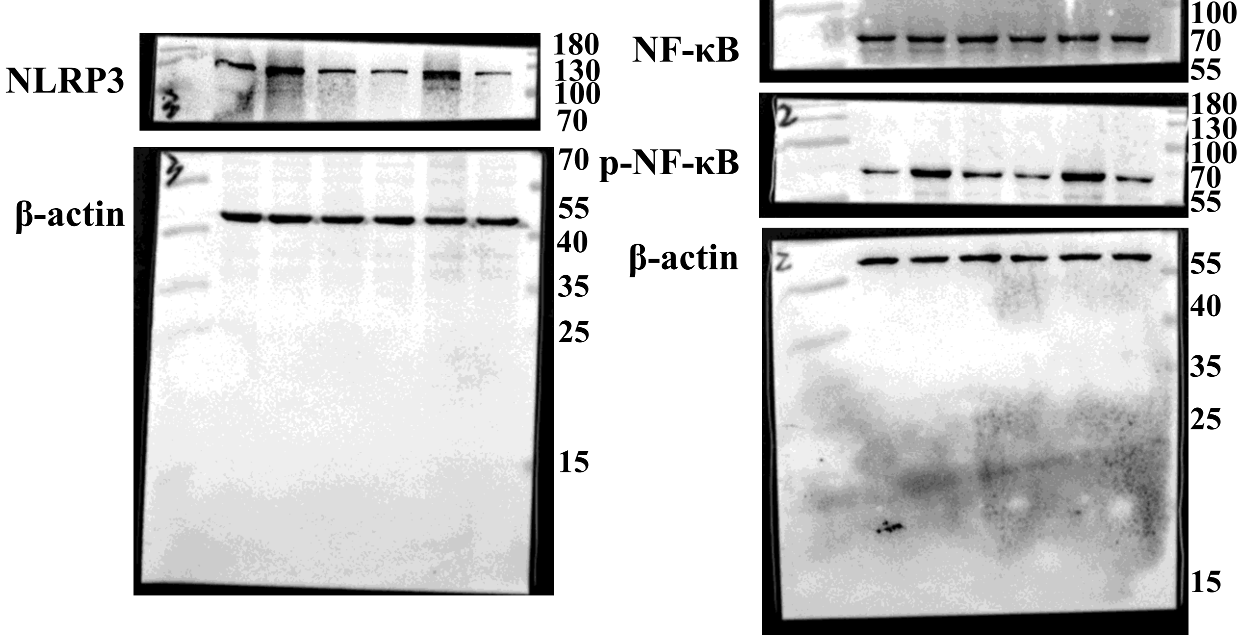

Fig. S7g

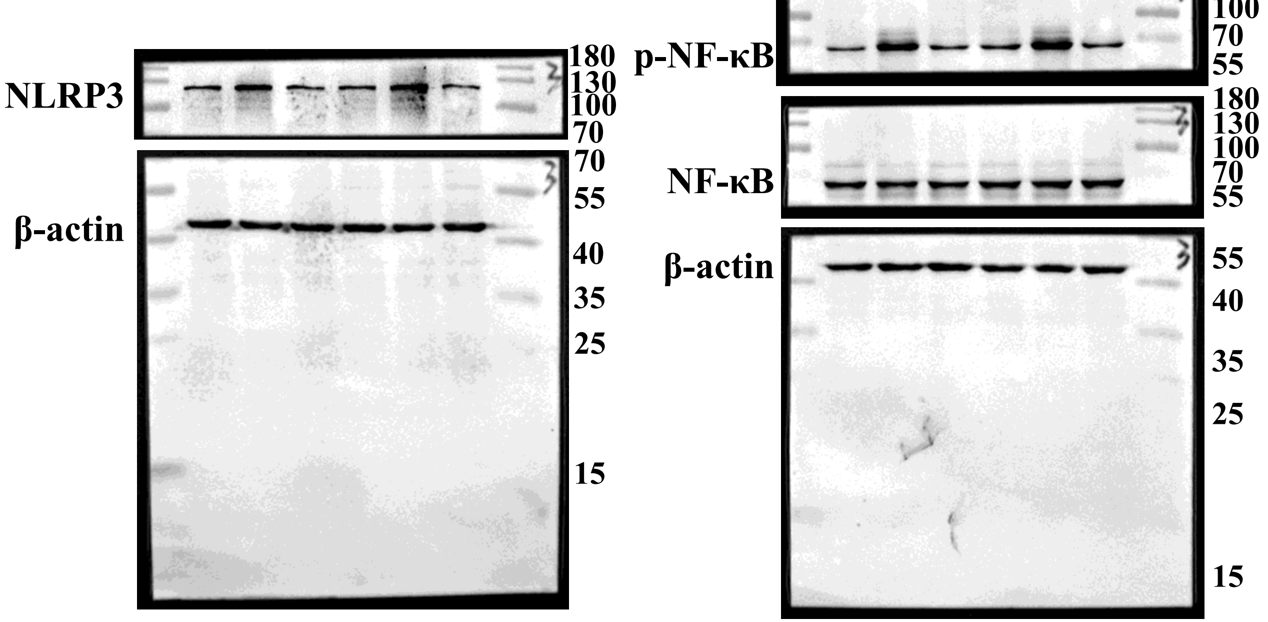

Fig. S7k

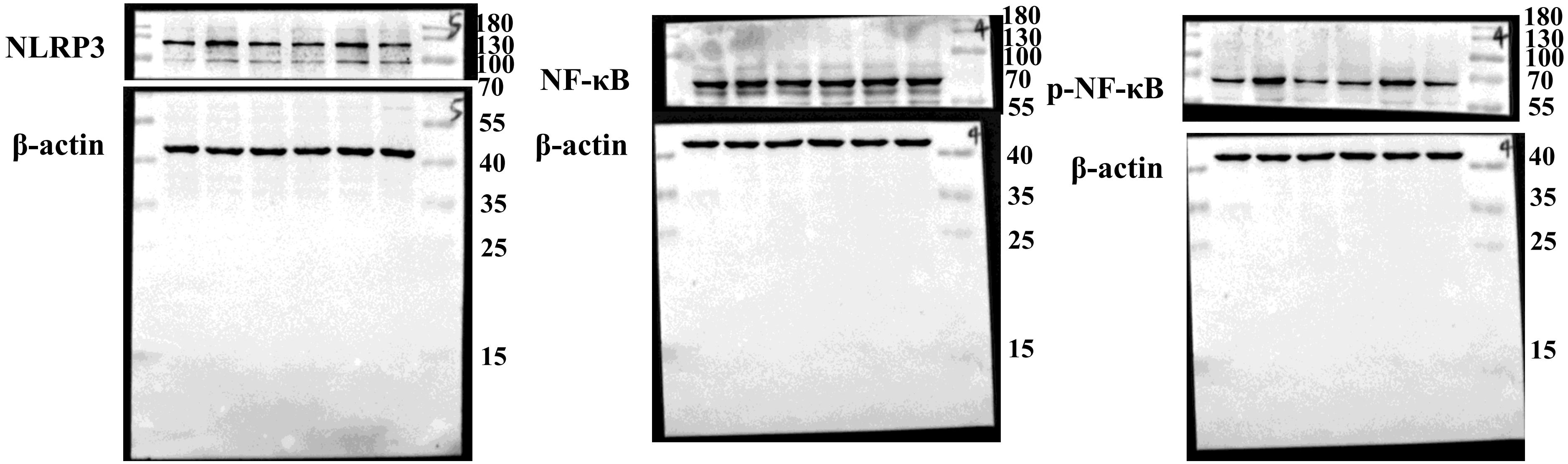

Supplement: Supplementary file 1 — Supplementary Material 1. [file 12974_2026_3845_MOESM1_ESM.pdf]
