# Supplementary material for: Otilonium bromide ameliorates paclitaxel-induced peripheral neuropathy by targeting phosphatase PPM1A
Source: J Neuroinflammation. 2026 May 7;23:224. doi: 10.1186/s12974-026-03845-9 (PMC13321745; doi:10.1186/s12974-026-03845-9)
Supplement: Supplementary file 2 — Supplementary Material 2. [file 12974_2026_3845_MOESM2_ESM.docx]

**Supplementary Materials**

**Supplementary Figures and Figure legends**

**
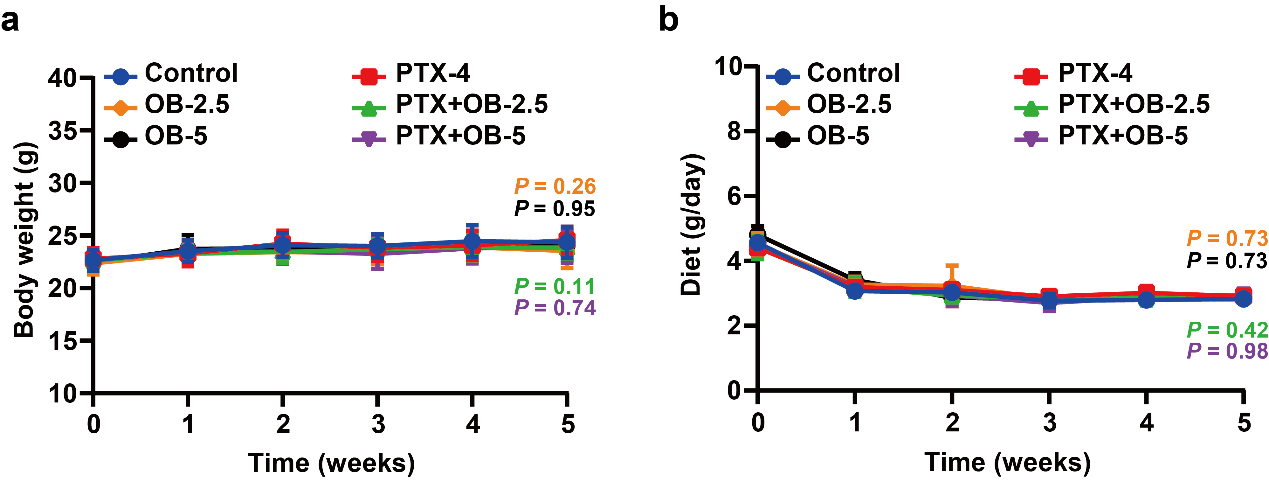
**

**Figure S1. OB did not affect the body weight and food intake in PIPN mice.** The **(a)** body weight and **(b)** food intake of PIPN mice treated with OB (*n* = 12 per group). All values are presented as mean ± SEM. Statistical significance was determined using one-way ANOVA followed by Dunnett’s post hoc test. Exact P values are shown above the bars.

**
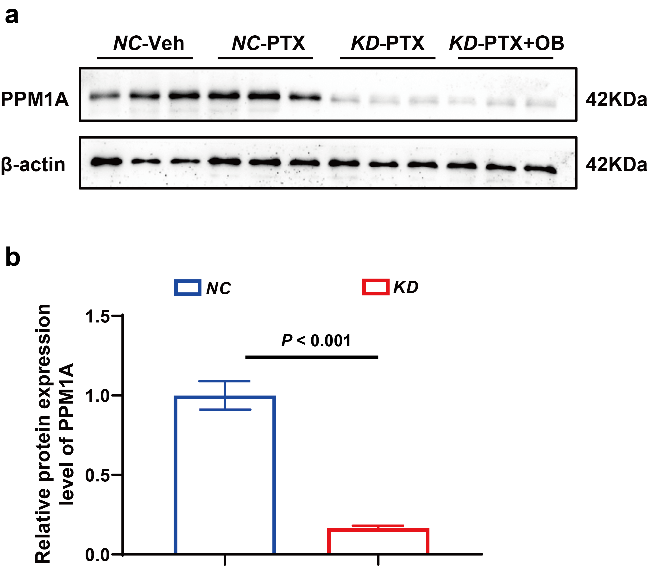
**

**Figure S2. Knockdown efficiency of PPM1A. (a, b)** Western blot analysis and quantification of PPM1A protein levels in DRG tissues of AAV-*Ppm1a-shRNA* injected PIPN mice (*n* = 6 per group). All values are presented as mean ± SEM. Statistical significance was determined using Student’s t-test. Exact P values are shown above the bars.

**
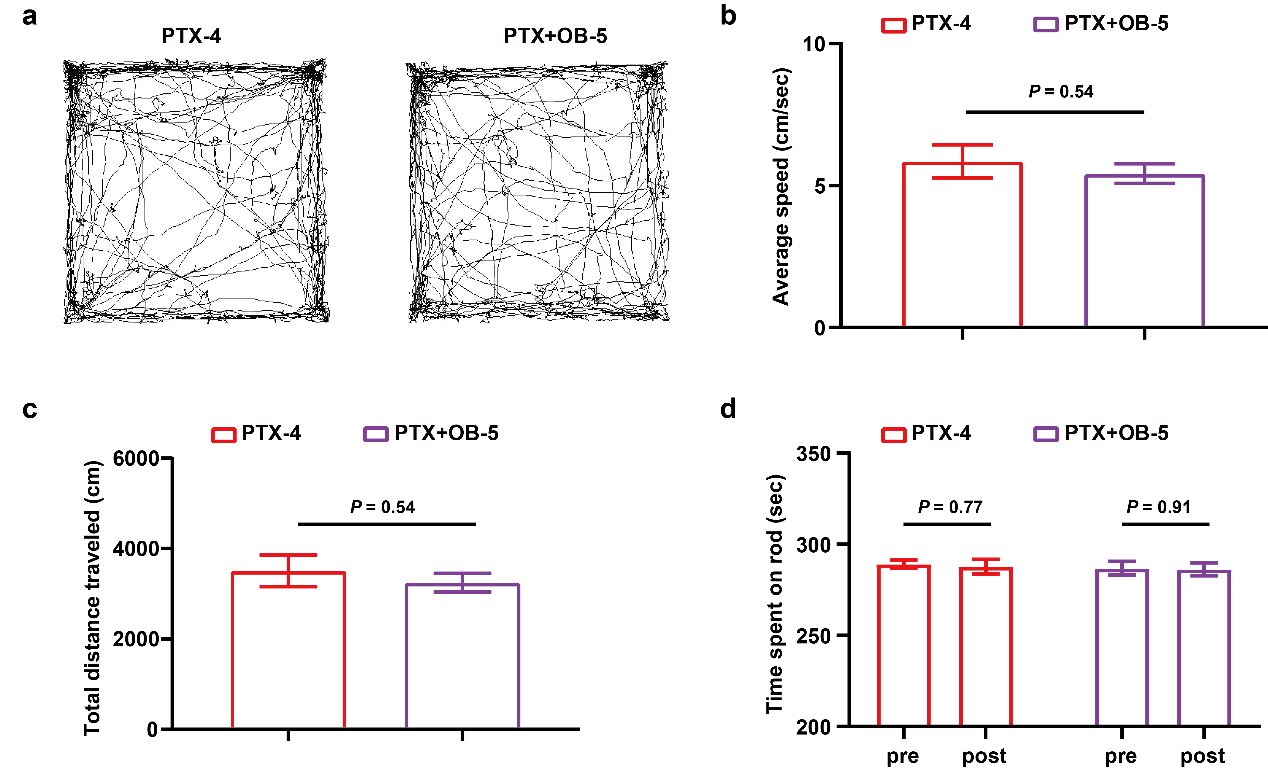
**

**Figure S3. OB treatment lacks CNS toxicity. (a)** Open field track plots of PIPN mice treated with vehicle or OB (*n* = 6 per group). **(b)** Total distance travelled by PIPN mice treated with vehicle or OB (*n* = 6 per group). **(c)** Average speed of PIPN mice in open field arena after treatment with vehicle or OB (*n* = 6 per group). **(d)** Time spent on the rotating rod of PIPN mice treated with vehicle or OB in the rotarod test (*n* = 6 per group). All values are presented as mean ± SEM. Statistical significance was determined using Student’s t-test. Exact P values are shown above the bars.

**
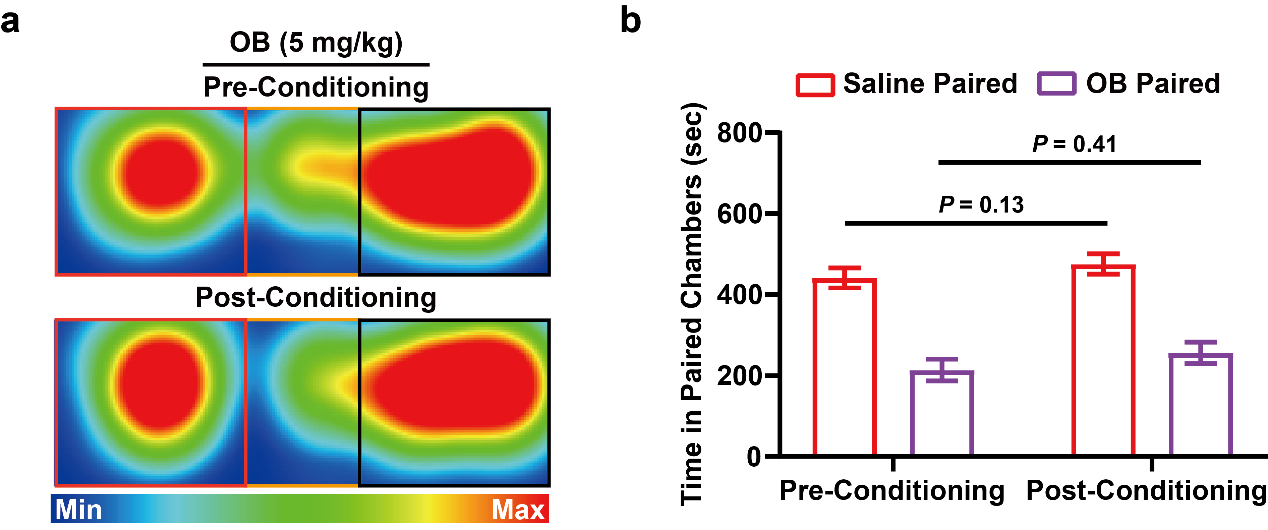
**

**Figure S4. OB treatment shows no impact on spontaneous pain. (a)** Heat maps recorded during pre-conditioning and post-conditioning with saline or OB (5 mg/kg) paired chambers (*n* = 6 per group). **(b)** Natural preference of PIPN mice (*n* = 6 per group). All values are presented as mean ± SEM. Statistical significance was determined using Student’s t-test. Exact P values are shown above the bars.

**
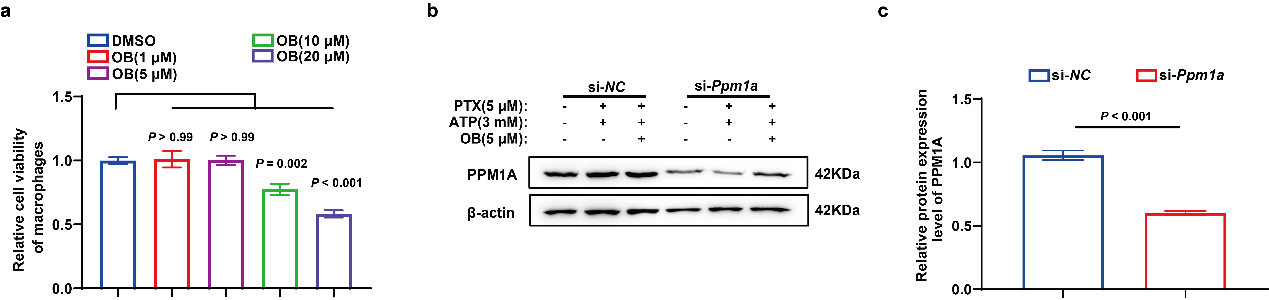
**

**Figure S5. Cytotoxicity of OB on macrophages and knockdown efficiency of PPM1A in macrophages. (a)** Macrophages were treated with OB at concentrations of 1, 5, 10 and 20 μM for 24 h and cell viability was tested by MTT assay (*n* = 4 per group). **(b, c)** Western blot analysis and quantification of PPM1A protein levels in macrophages transfected with siRNA (*n* = 3 per group). All values are presented as mean ± SEM. Statistical significance was determined using one-way ANOVA with Dunnett’s *post hoc* test and Student’s t-test. Exact P values are shown above the bars.

**
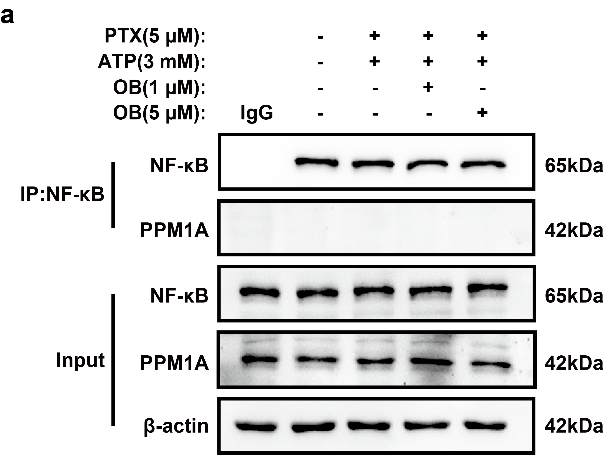
**

**Figure S6. PPM1A does not directly interact with NF-κB. (a)** Co-IP and Western blot analysis of PPM1A and NF-κB in macrophages (*n* = 3).

**
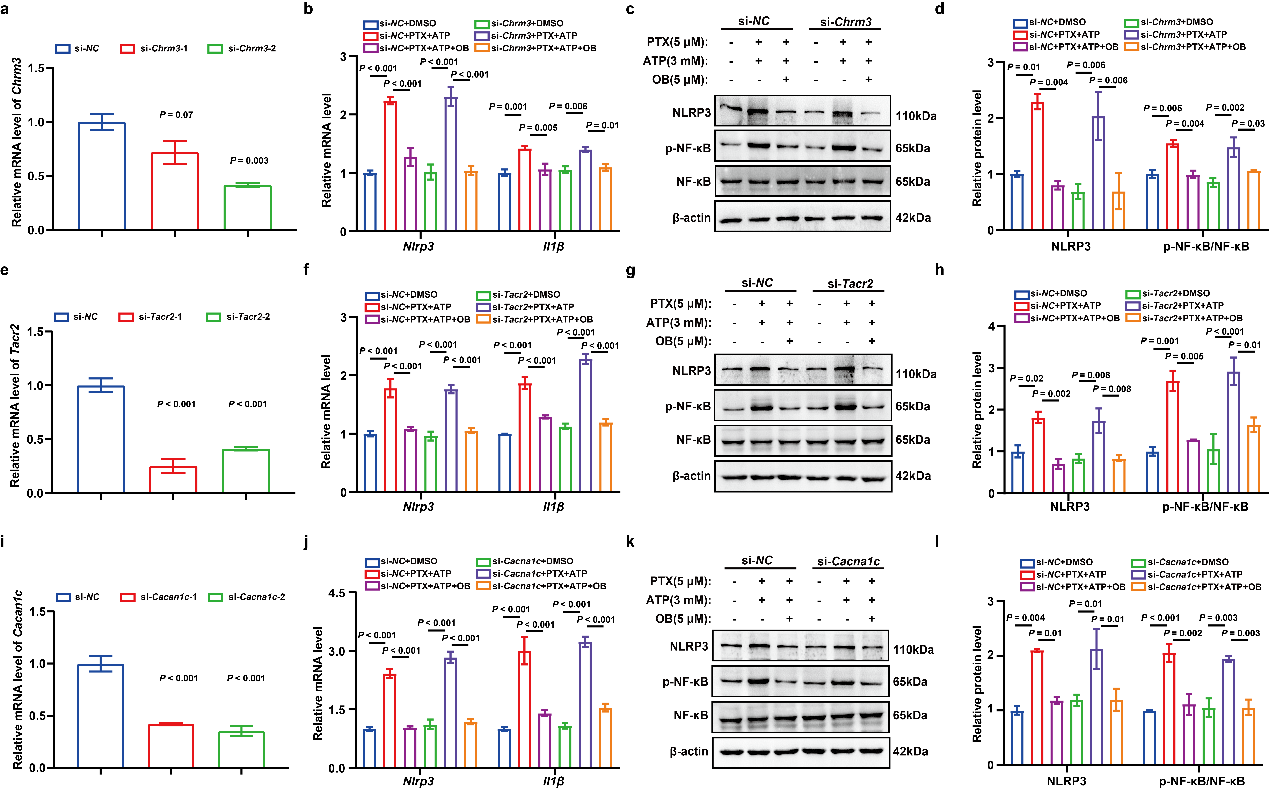
**

**Figure S7. OB exerts its anti-inflammatory effects independently of traditional anti-spasmodic targets. (a)** qPCR analysis of *Chrm3* mRNA level in macrophages transfected with si-*Chrm3* (*n* = 3 per group). **(b)** qPCR analysis of *Nlrp3* and *Il1β* mRNA levels in macrophages transfected with si-*Chrm3* (*n* = 3 per group). **(c, d)** Western blot analysis and quantification of NLRP3, p-NF-κB and NF-κB protein levels in macrophages transfected with si-*Chrm3* (*n* = 3 per group). **(e)** qPCR analysis of *Tacr2* mRNA level in macrophages transfected with si-*Tacr2* (*n* = 3 per group). **(f)** qPCR analysis of *Nlrp3* and *Il1β* mRNA levels in macrophages transfected with si-*Tacr2* (*n* = 3 per group). **(g, h)** Western blot analysis and quantification of NLRP3, p-NF-κB and NF-κB protein levels in macrophages transfected with si-*Tacr2* (*n* = 3 per group). **(i)** qPCR analysis of *Cacna1c* mRNA level in macrophages transfected with si-*Cacna1c* (*n* = 3 per group). **(j)** qPCR analysis of *Nlrp3* and *Il1β* mRNA levels in macrophages transfected with si-*Cacna1c* (*n* = 3 per group). **(k, l)** Western blot analysis and quantification of NLRP3, p-NF-κB and NF-κB protein levels in macrophages transfected with si-*Cacna1c* (*n* = 3 per group). All values are presented as mean ± SEM. Statistical significance was determined using one-way ANOVA followed by Dunnett’s post hoc test. Exact P values are shown above the bars.

**
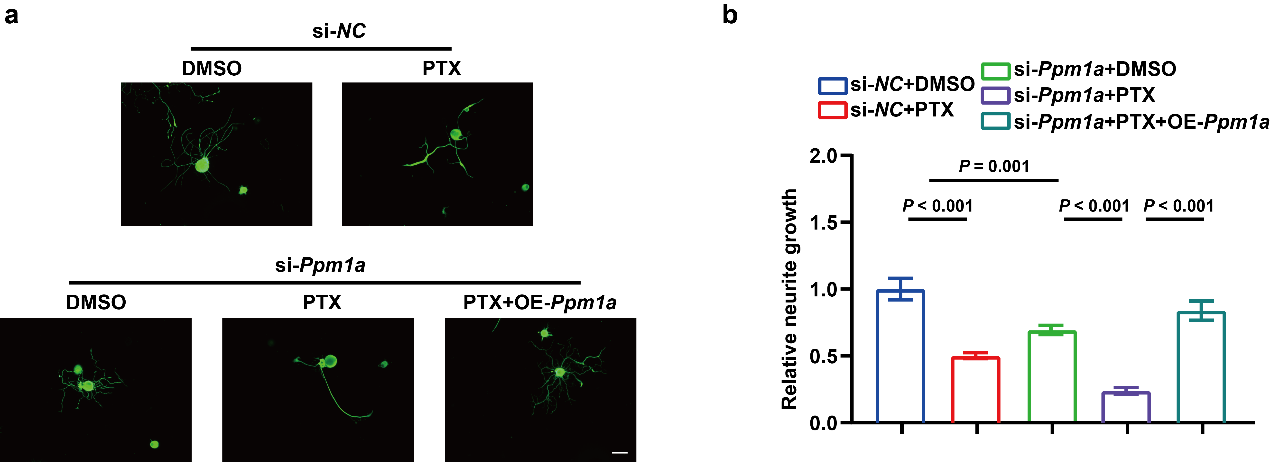
**

**Figure S8.** **Overexpression of PPM1A restores PTX-induced neurite length inhibition in DRG neurons transfected with si-*Ppm1a*.** Primary DRG neurons transfected with si-*Ppm1a* were treated with DMSO or 0.1 μM PTX for 24 h to induce neurite injury, followed by transfection with NC or *Ppm1a* overexpression plasmid (OE-*Ppm1a*) for 36 h. **(a, b)** Representative immunofluorescence images with quantification of total neurite length in primary DRG neurons (labeled by β-tubulin III, green) (*n* = 6 per group). Scale bars: 50 μm. All values are presented as mean ± SEM. Statistical significance was determined using one-way ANOVA followed by Dunnett’s post hoc test. Exact P values are shown above the bars.

**
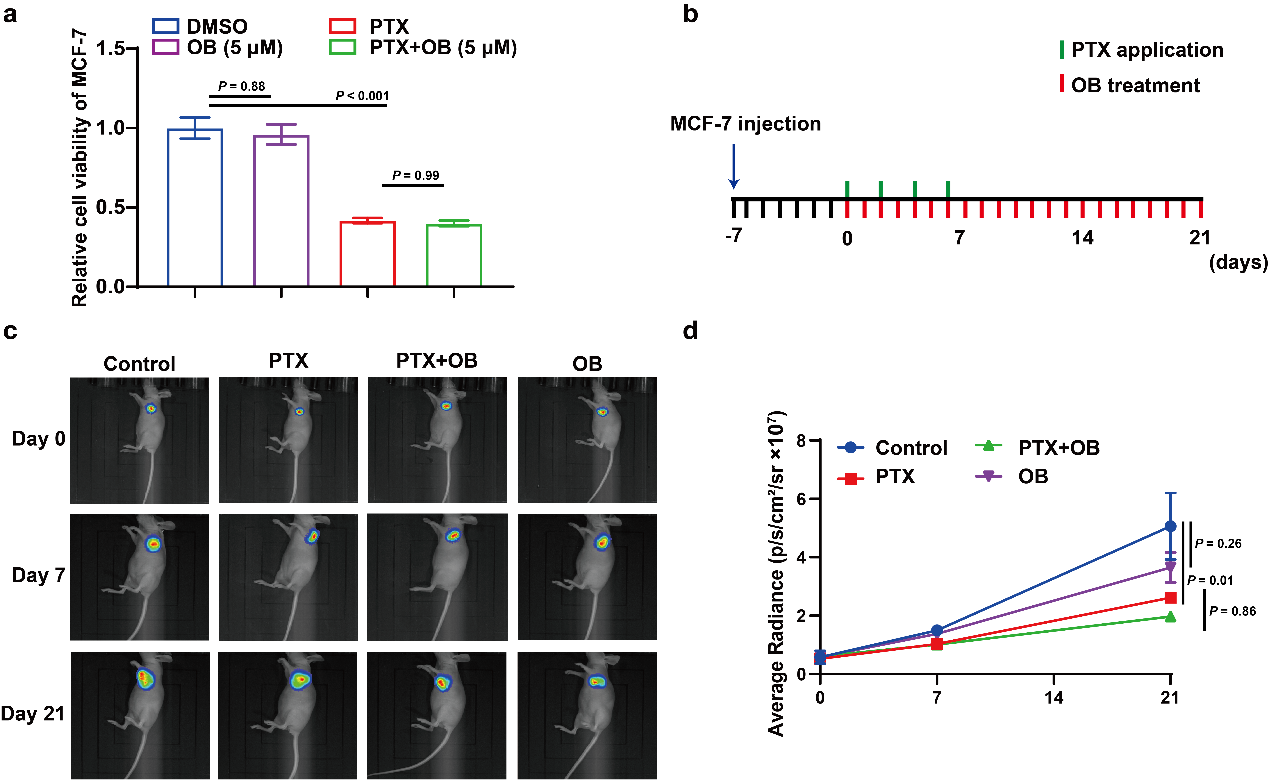
**

**Figure S9. OB does not affect the antitumor effect of PTX. (a)** MCF-7 cells were treated with OB (5 μM) or PTX (0.1 μM) for 24 h. Cell viability was tested by MTT assay (*n* = 5 per group). **(b)** Schedule of the in vivo experiment. **(c)** Representative bioluminescence images of tumors treated with PTX (4 mg/kg) or OB (5 mg/kg) (*n* = 3 per group). **(d)** Quantification results for **(c)**. All values are presented as mean ± SEM. Statistical significance was determined using one-way ANOVA followed by Dunnett’s *post hoc* test. Exact P values are shown above the bars.

**Supplementary Tables**

**Table S1. The siRNA and shRNA oligonucleotides sequence.**

| **genes** | **Sense (5’-3’)** | **Anti-Sense (5’-3’)** |
| --- | --- | --- |
| si-*Ppm1a* | GCAGAUAGAAGCGGGUCAATT | UUGACCCGCUUCUAUCUGCTT |
| si-*Chrm3* | GGCAGUUCUCGAAGCUGUAGC | UACAGCUUCGAGAACUGCCUG |
| si-*Tacr2* | GCAAGAUGCUCCUACUGUAUC | UACAGUAGGAGCAUCUUGCCU |
| si-*Cacna1c* | GACUAUGUCUUCACUAGUAUC | UACUAGUGAAGACAUAGUCUG |
| sh-*Ppm1a* | GCAGATAGAAGCGGGTCAATTCAAGAGATTGACCCGCTTCTATCTGCTTTTTT | |

**Table S2. The primary antibodies used for immunostaining assay.**

| **Antibody** | **Dilution ratio** | **Source and identifier** |
| --- | --- | --- |
| anti-PGP9.5 | 1:500 | Abcam, ab108986 |
| anti-MBP | 1:500 | Sigma-Aldrich, ab7349 |
| anti-β tubulin III | 1:1000 | Sigma-Aldrich, T8578 |
| anti-PPM1A | 1:500 | Abcam, ab14824 |
| anti-NLRP3 | 1:500 | Invitrogen, PA5-79740 |
| anti-ASC | 1:500 | CST, 67824S |
| anti-IL-1β | 1:500 | CST, 12242S |
| anti-NF-κB P65 | 1:500 | CST, 8242S |
| anti-CD68 | 1:500 | Santa Cruz, sc-20060 |
| anti-TNF-α | 1:500 | CST, 11948S |
| anti-CD206 | 1:500 | Santa Cruz, sc58986 |

**Table S3. Specific primer sequences**

| **Gene** | **Primer** | **Sequence** |
| --- | --- | --- |
| *Il1β* | CCTACTTCAGCATCCTCTACTGG | AGGGTTTCTTGAGAAGGGGAC |
| *Arg1* | CATTGGCTTGCGAGACGTAGAC | GCTGAAGGTCTCTTCCATCACC |
| *Cd86* | TCAGTCAGGATGGGAGTGGTA | ATCCAAGAGCCATTCCTACCT |
| *Cd206* | GTTCACCTGGAGTGATGGTTCTC | AGGACATGCCAGGGTCACCTTT |
| *Il6* | TCTTGGGACTGATGCTGGTG | CAGAATTGCCATTGCACAACTC |
| *Il10* | AGGGCACCCAGTCTGAGAACA | CGGCCTTGCTCTTGTTTTCAC |
| *Tnfα* | CCAGACCCTCACACTCAGATC | ATAGCAAATCGGCTGACGGT |
| *Nlrp3* | ATTACCCGCCCGAGAAAGG | TCGCAGCAAAGATCCACACAG |
| *Ppm1a* | CGCACGTTAGCCAGTGAGAA | CGCAGAATCAGTGTCGTCATT |
| *Hmgb1* | GGCGAGCATCCTGGCTTATC | GGCTGCTTGTCATCTGCTG |
| *Chrm3* | ACTATGTGGCCAGCAATGCT | CGGCTCGTTTTGTTGTTCGT |
| *Tacr2* | GGCCTTGAGAGTAACGCAACA | GGCCAGAATGATCCAGATGACT |
| *Cacna1c* | TGAACTCAGTGCGCTCCATT | TCCTACGGGTCTGCATCTCA |
| *β-actin* | GGCTGTATTCCCCTCCATCG | CCAGTTGGTAACAATGCCATGT |

**Table S4. The primary antibodies used for Western blot.**

| **Antibody** | **Dilution ratio** | **Source and identifier** |
| --- | --- | --- |
| anti-PPM1A | 1:1000 | Abcam, ab14824 |
| anti-p-Smad3 | 1:1000 | CST, 9520S |
| anti-Smad3 | 1:1000 | CST, 9523S |
| anti-NLRP3 | 1:1000 | CST, 15101S |
| anti-ASC | 1:1000 | CST, 67824S |
| anti-IL-1β | 1:1000 | CST, 12242S |
| anti-β-actin | 1:1000 | Absin, ABS830031SS |
| anti-Caspase1 P20 | 1:1000 | Adipogen, AG-20B-0042 |
| anti-p-NF-κB P65 | 1:1000 | CST, 3033S |
| anti-NF-κB P65 | 1:1000 | CST, 8242S |
| anti-IKKβ | 1:1000 | Wanleibio, WLA0347 |
| IKKβ | 1:1000 | CST, 2678S |
